# Cyclic AMP Pathway Suppress Autoimmune Neuroinflammation by Inhibiting Functions of Encephalitogenic CD4 T Cells and Enhancing M2 Macrophage Polarization at the Site of Inflammation

**DOI:** 10.3389/fimmu.2018.00050

**Published:** 2018-01-25

**Authors:** Tatyana Veremeyko, Amanda W. Y. Yung, Marina Dukhinova, Inna S. Kuznetsova, Igor Pomytkin, Alexey Lyundup, Tatyana Strekalova, Natasha S. Barteneva, Eugene D. Ponomarev

**Affiliations:** ^1^School of Biomedical Sciences, The Chinese University of Hong Kong, Shatin, Hong Kong; ^2^Department of Advanced Cell Technologies, Institute for Regenerative Medicine, I.M. Sechenov First Moscow State Medical University, Moscow, Russia; ^3^Department of Neuroscience, Maastricht University, Maastricht, Netherlands; ^4^Laboratory of Psychiatric Neurobiology, Institute of Molecular Medicine, I.M. Sechenov First Moscow State Medical University, Moscow, Russia; ^5^Department of Pediatrics, Program in Cellular and Molecular Medicine, Children’s Hospital Boston, Harvard Medical School, Boston, MA, United States; ^6^School of Science and Technology, Nazarbayev University, Astana, Kazakhstan

**Keywords:** Th1 cells, neuroinflammation, Forskolin, cAMP, microRNA-124, macrophage, M2 polarization, ERK

## Abstract

Although it has been demonstrated that cAMP pathway affect both adaptive and innate cell functions, the role of this pathway in the regulation of T-cell-mediated central nervous system (CNS) autoimmune inflammation, such as in experimental autoimmune encephalomyelitis (EAE), remains unclear. It is also unclear how cAMP pathway affects the function of CD4 T cells *in vivo* at the site of inflammation. We found that adenylyl cyclase activator Forskolin besides inhibition of functions autoimmune CD4 T cells also upregulated microRNA (miR)-124 in the CNS during EAE, which is associated with M2 phenotype of microglia/macrophages. Our study further established that in addition to direct influence of cAMP pathway on CD4 T cells, stimulation of this pathway promoted macrophage polarization toward M2 leading to indirect inhibition of function of T cells in the CNS. We demonstrated that Forskolin together with IL-4 or with Forskolin together with IL-4 and IFNγ effectively stimulated M2 phenotype of macrophages indicating high potency of this pathway in reprogramming of macrophage polarization in Th2- and even in Th1/Th2-mixed inflammatory conditions such as EAE. Mechanistically, Forskolin and/or IL-4 activated ERK pathway in macrophages resulting in the upregulation of M2-associated molecules miR-124, arginase (Arg)1, and Mannose receptor C-type 1 (Mrc1), which was reversed by ERK inhibitors. Administration of Forskolin after the onset of EAE substantially upregulated M2 markers Arg1, Mrc1, Fizz1, and Ym1 and inhibited M1 markers nitric oxide synthetase 2 and CD86 in the CNS during EAE resulting in decrease in macrophage/microglia activation, lymphocyte and CD4 T cell infiltration, and the recovery from the disease. Forskolin inhibited proliferation and IFNγ production by CD4 T cells in the CNS but had rather weak direct effect on proliferation of autoimmune T cells in the periphery and *in vitro*, suggesting prevalence of indirect effect of Forskolin on differentiation and functions of autoimmune CD4 T cells *in vivo*. Thus, our data indicate that Forskolin has potency to skew balance toward M2 affecting ERK pathway in macrophages and indirectly inhibit pathogenic CD4 T cells in the CNS leading to the suppression of autoimmune inflammation. These data may have also implications for future therapeutic approaches to inhibit autoimmune Th1 cells at the site of tissue inflammation.

## Introduction

Various pathways are known to affect differentiation and function of CD4 T cells *in vivo* during inflammation associated with autoimmunity or infection. One of most common and important pathways in the process is cAMP pathway that is known to be involved in negative regulation of T cell activation and proliferation (1). However, more detailed and recent studies demonstrated that cAMP-inducing agents *Cholera Toxin*, 8-bromo-cAMP, and FDA-approved drug Forskolin inhibited proliferation of Th1 but not Th2 effector cells *in vitro* (2). In addition, it was shown that *Cholera Toxin*-induced expansion rather than inhibition of Th17 cells *in vivo* (3). *Pertussis toxin*, which was specifically used for active induction of central nervous system (CNS) autoimmune inflammation, was also shown to activate adenylyl cyclase leading to increase in cAMP (4). However, *Pertussis toxin* stimulated rather than inhibited expansion of Th1 cells *in vivo* leading to development of CNS autoimmune inflammation (5). Moreover, selective inhibition of cAMP pathway *in vivo* in CD4 T cells demonstrated that cAMP was required for differentiation and proliferation of Th1 and Th17 cells but not Th2 and Tregs (6). Thus, exact role of cAMP pathway in the modulation of function of effector T cells during CNS autoimmune inflammation remains unclear. An important factor that could affect functions of T cells in the tissues during inflammation are tissue-resident and blood-derived macrophages that are recruited to the sited of inflammation and could be also affected by cAMP-inducing agents.

During inflammation, macrophages become activated under the influence of T-cell-derived cytokines or pathogens leading to two or more distinct (polarized) states. Polarization of macrophages toward the classic M1 phenotype is induced by Th1 cytokines such as IFNγ and the alternative M2 phenotype induced by Th2 cytokines such as IL-4 plays an important role in regulation of T cells functions during infection and autoimmune diseases (7). Recently, it was suggested that macrophages do not form stable populations, but rather have distinct phenotypes in response to various inflammatory stimuli (e.g., IFNγ vs. IL-4) and often form mixed phenotypes (7, 8), which has unpredictable impact on functions of T cells at the site of inflammation where macrophages serve as antigen-presenting cells. In normal conditions, the CNS has specific microenvironment where CNS-resident macrophages (also referred to as microglia) have intrinsic M2-like phenotype and express number of M2 markers (e.g., Ym1 and IL-4) and specific microRNAs (miRs) (e.g., miR-124) that promote M2 polarization (9–11). Moreover, CNS has internal source of IL-4, which plays critical role in suppression of neuroinflammation such as experimental autoimmune encephalitis (EAE) (9). M2 macrophages in normal CNS express low level of MHC class II and CD86 and do not effectively stimulate T cells.

Similar to human disease multiple sclerosis (MS), EAE is an inflammatory disease of the CNS that is initiated by autoimmune Th1 and Th17 cells that recognize self-antigen within myelin sheath [e.g., myelin oligodendrocyte glycoprotein (MOG)] (12). Th1 and Th17 cells produce number of cytokines such as IFNγ, TNF, and GM-CSF that mediate M1 polarization of macrophages that comprise ~70% of CNS inflammatory lesions and mediate most of the damage of neuronal tissue by producing TNF, nitric oxide (NO), etc. (7, 12–15). Since CNS has intrinsic M2-skewing microenvironment, macrophages in the CNS during EAE and other pathologies often exhibit mixed M1/M2 phenotype under the influence of CNS-derived IL-4 and Th1-derived IFNγ (16). We have previously found that during EAE macrophages exhibit dually activated phenotype expressing both M1 and M2 markers such as CD86 and Ym1 at the same time (9). These results were not surprising, since during EAE both M1 (IFNγ) and M2 (IL-4) cytokines were present in inflamed CNS. Therefore, it is important to find new pathways and co-factors that could skew macrophage polarization toward M2 in mixed inflammatory conditions during EAE.

One of the co-factors that help to skew macrophages toward M2 phenotype in the CNS is miR-124 (10). miRs are class of short (20–22 nucleotides) non-coding RNAs that regulate many physiological processes including macrophages polarization (11). We have previously shown that miR-124 is expressed in microglia in normal CNS contributing to M2 phenotype of microglial cells in resting state (10). During EAE, expression of miR-124 in activated microglia was decreased, while transfection of macrophages with miR-124 upregulated M2 markers such as arginase (Arg)1 and Mannose receptor C-type 1 (Mrc1) and downregulated M1 markers such as CD86, nitric oxide synthetase (NOS)2, and TNF leading to recovery from the disease (10). We also found that miR-124 is upregulated in bone marrow (BM)-derived and peritoneal macrophages in response to IL-4, but effect of IL-4 on upregulation of miR-124 was modest (17). Moreover, expression of miR-124 in microglia in normal CNS was IL-4/IL-13 independent, while IFNγ pretreated M1 macrophages failed to upregulate miR-124 in response to subsequent treatment with IL-4 (17). These data suggest the importance of other pathways that may stimulate miR-124 and change balance toward M2 in the CNS during inflammation.

Forskolin (coleonol) originate from Indian plant Coleus and currently used as food supplement or as a drug for traditional oriental medicine and anticancer therapy (18). We found that administration of Forskolin during EAE inhibited proliferation and IFN-γ production by CD4 T cells in the CNS and upregulated a number of M2-associated molecules miR-124, Arg1, Mrc1, Ym1, and Fizz1 and downmodulated M1-associated molecules MHC class II, CD86, and NOS2. This indicates very potent effect of this drug in the modulation of autoimmune CD4 T cells and macrophage activation/polarization at the site of tissue inflammation. To understand mechanisms, we were looking for pathways that inhibit pathogenic CD4 T cells, enhance action of IL-4 in M2 polarization and upregulation of miR-124 during CNS autoimmune inflammation. We found that Forskolin did not directly affect CD4 T cells, but promoted M2 polarization of macrophages. Further analysis demonstrated that Forskolin and/or IL-4 activated cAMP-responsive element-binding/activating transcription factor (CREB/ATF) family proteins that contributed to upregulation of miR-124 and M2 polarization.

## Materials and Methods

### Mice

C57BL/6 mice were originally purchased from the Jackson Laboratory and bred locally at the Laboratory Animal Services Center at the Chinese University of Hong Kong. All animal procedures were conducted under individual licenses from Hong Kong government and approved by animal ethics committee form the Chinese University of Hong Kong.

### Cells

Bone marrow-derived macrophages were grown in DMEM media (Gibco) supplemented with 10% FBS (Gibco) and M-CSF (R&D; 10 ng/ml) for 5 days as described (10) and used for analysis. Peritoneal macrophages were isolated by peritoneal lavage and removal of non-adhered cells after 14–16 h of incubation at CO_2_ incubator (9). Flow cytometry analysis indicated 96–98% of CD11b^+^F4/80^+^ cells in case of BM-derived and peritoneal macrophages. For macrophage polarization, the cells were treated with IL-4 (50 ng/ml), IFNγ (100 ng/ml), and/or Forskolin (30 μM; Sigma) for indicated periods ranging from 2 to 24 h. The optimal dosage of Forskolin was determined after performing dose–response curve (1–100 μM) for the upregulation of miR-124. ERK1/2 inhibitor U0126 was purchased from Cell Signaling, and another ERK1/2 PD184352 inhibitor was purchased from Abcam. Inhibitors U0126 and PD184352 were used at concentrations of 10 μM and 200 nM, respectively, according to the manufacturer’s recommendations. Taurolidin (Sigma) was used at the dose of 100 μM. X5050 inhibitor was used at final concentration of 100 μM. NRSE inhibitory dsRNA was used at concentration of 20 nM as described (19). Primary cultures of mouse cortical neurons were performed as described earlier (10).

### EAE Induction

EAE was induced by subcutaneous immunization with 150 μg MOG_35–55_ (American peptide) in 4 mg/ml CFA (Diffco) as described earlier (10). Pertussis toxin (Sigma) was injected i.p. (150 ng/mouse) on day 0 and day 2 post-immunization. Individual animals were assessed daily for symptoms of EAE and scored using a scale from 0 to 5 as follows: 0, no disease; 0.5, weak tail or mild hind limb ataxia; 1, limp tail and/or hind limb ataxia; 2, hind limb paresis; 3, hind limb paralysis; 4, hind and fore limb paralysis; 5, death. Forskolin (3 mg/kg) or vehicle (PBS with 1% DMSO) was injected i.p. daily starting from disease onset (day 14) till the peak of disease (days 21–22). The optimal dosage of Forskolin for *in vivo* administration was selected by titrating down reported dosage of 4–5 mg/kg in previously published study for anticancer therapy in mouse model (20).

### Real-time PCR

For quantitation of various markers and molecules by real-time RT PCR, total RNA was isolated by Qiagene or MirVana (Applied Biosystem) kits from cultured BM-derived, or peritoneal macrophages, or form the spinal cords of PBS-perfused unmanipulated mice or mice on day 22 following EAE. For analysis of miRNA expression, real-time RT-PCR analyses were performed using TaqMan miRNA assays for miR-124 (Applied Biosystems) and relative expressions were calculated using the Δ*C*_T_ method and normalized to uniformly expressed snoRNA55 (Applied Biosystems) as we described earlier (10, 21). For analysis of mRNA expression for NOS2 (forward primer, 5′-ACCCACATCTGGCAGAATGAG-3′; reverse primer, 5′-AGCCATGACCTTTCGCATTAG-3′), Arg1 (forward primer, 5′-CTTGGCTTGCTTCGGAACTC-3′; reverse primer, 5′-GGAGAAGGCGTTTGCTTAGTTC-3′), and Fizz1 (forward primer, 5′-GCCAGGTCCTGGAACCTTTC-3′; reverse primer, 5′-GGAGCAGGGAGATGCAGATGAG-3′), ATF3 forward 5′-AGCATTCACACTCTCCAGTTTCTCT-3′, reverse 5′-GGAGAAGACAGAGTGCCTGC-3′, IL-1β forward 5′-CTTCCAGGATGAGGACATGAGCAC-3′, reverse 5′-TCATCATCCCATGAGTCACAGAGG-3′, IL-6 forward 5′-CCTTCTTGGGACTGATGCTGGTG-3′, TNF forward 5′-AGCCGATGGGTTGTACCTTG-3′ reverse 5′-GTGGGTGAGGAGCACGTAGTC-3′, Mrc1 forward 5′-ACCACGGATGACCTGTGCTC-3′ reverse 5′-TGGTTCCACACCAGAGCCATC-3′, CCDN1 forward 5′-GCAGACCATCCGCAAGCATG-3′, reverse 5′-TGGAGGGTGGGTTGGAAATGAAC-3′, RE1-silencing transcription factor (REST) (for alternative splicing) forward 5′-CGACACATGCGGACTCATTC-3′, reverse 5′-GCATGTCGGGTCACTTCATG-3′; Ym1 forward 5′-CCATTGGAGGATGGAAGTTTG-3′, reverse 5′-GACCCAGGGTACTGCCAGTC-3′ the relative expressions were calculated using the Δ*C*_T_ method and normalized to the GADPH housekeeping gene (forward primer, 5′-ATGACCACAGTCCATGCCATC-3′; reverse primer, 5′-GAGCTTCCCGTTCAGCTCTG-3′), and then relative level of expression was calculated in comparison to control conditions as we did earlier (10).

### Western Blotting

Western blotting analysis was performed according to standard protocol as we reported previously (10). Antibodies for ERK, phospho-ERK, CEBP, phospho-CEBP, CREB, phospho-CREB, REST, and β-actin were purchased from Cell Signaling.

### Flow Cytometry

Mononuclear cells were isolated from the CNS or spleens of mice with EAE on day 22 using Percoll gradients as described in our previous studies (13, 21). For analysis of microglia, macrophages, and lymphocyte populations and macrophage/microglia activation status, the cells were stained with anti-CD11b-AF480, anti-CD86-PE, anti-MHC class II-PE-Cy5 (all from BD Bioscience), and F4/80-APC (eBiosceince) or anti-CD45-APC-Cy7 (BioLegend). FcRs were blocked with a mAb specific for mouse FcR (2.4G2; BD Biosciences). CD4 T cells were stained for analysis of surface markers with anti-CD4-APC, or anti-CD4-APC and anti-TCRβ-PE, or anti-CD4-APC and anti-TCR-Vβ_11_-PE (all from BD Biosciences). These data were acquired on LSR Fortessa cytometer (BD Biosciences) and analyzed using FlowJo software (TreeStar Inc.). Absolute numbers of the cells were calculated by counting total number of mononuclear cells using hemocytometer and by multiplying by the percentages of particular populations obtained by flow cytometry.

### T-Cell and Macrophage Proliferation Assay

BrdU incorporation assay was used to assess proliferation of BM-derived macrophages or CD4 T cells *in vitro* or *in vivo* similar as was described in our earlier studies (12). To perform this analysis, we used BrdU kit from BD Biosciences following the manufacturer’s instructions. BrdU was injected intraperitoneally (2 mg/mouse) or added to cultures at final concentration 10 μM 14 h before analysis. Macrophages were stained for cell surface markers CD11b, F4/80, and for intracellular BrdU-FITC (BD Biosciences) and CD11b^+^F4/80^+^ gated analyzed for BrdU incorporation by three-color flow cytometry. CD4 T cells were stained for cell surface markers CD4 alone, or CD4 and TCR-Vβ_11_, and intracellular BrdU and analyzed by two- or three-color flow cytometry.

### Cytokine Production by CD4 T Cells

For intracellular detection of IFNγ expression, *ex vivo* isolated or cultured CNS or splenic mononuclear cells were activated with phorbol myristate acetate (50 ng/ml) and ionomycin (1 μg/ml; both from Sigma) in the presence of GolgiStop (1 μl/ml, BD Biosciences) for 4 h. Cells were washed, immediately stained for surface marker anti-CD4-APC, fixed/permeabilized using BD intracellular cytokine detection kit, and further stained for intracellular IFNγ using anti-IFNγ-PE (all reagents were purchased from BD Biosciences). CD4^+^ gated cells were analyzed by two-color flow cytometry.

### T-Cell Recall Response

For CD4 T cell recall response, mice were immunized with 150 μg MOG_35–55_ (American peptide) in 4 mg/ml CFA (Diffco) as described earlier (10, 22). On day 7 post-immunization, CD4 T cells were isolated from draining lymph nodes and spleen using negative selection and magnetic beads and incubated in the presence of irradiated splenocytes, MOG_35–55_ peptide (5–10 μg/ml) or stimulating anti-CD3 (BD Biosciences, clone 145-2C11) and anti-CD28 (BD Biosciences, clone 37.51) mAbs as described earlier (12). The experiments were performed in the presence of 30 μM of Forskolin or 0.1% DMSO in media (Control). After 72 h of incubation, the dead cells were removed using Ficoll™ gradient centrifugation, washed and stained for cells surface markers CD4 and TCRVβ_11_, and were analyzed for BrdU incorporation by three-color flow cytometry.

### Immunohistochemistry

Fixed frozen section of lumbar areas of spinal cord were prepared and stained with luxol fast blue (LFB, Sigma) as we described earlier (10).

### Statistical Analysis

The results are presented as mean ± SE. Unpaired Student’s *t*-test was used to determine significance between two independent groups. *p* Values of less than 0.05 were considered to be significant. SigmaPlot program was used for the creation of the graphs and performing statistical analysis.

## Results

### Forskolin Downregulate MHC Class II and CD86 on Microglia and Macrophages in the CNS during EAE Resulting in Decrease of Neuroinflammation and Recovery from the Disease

**Figure 1:**
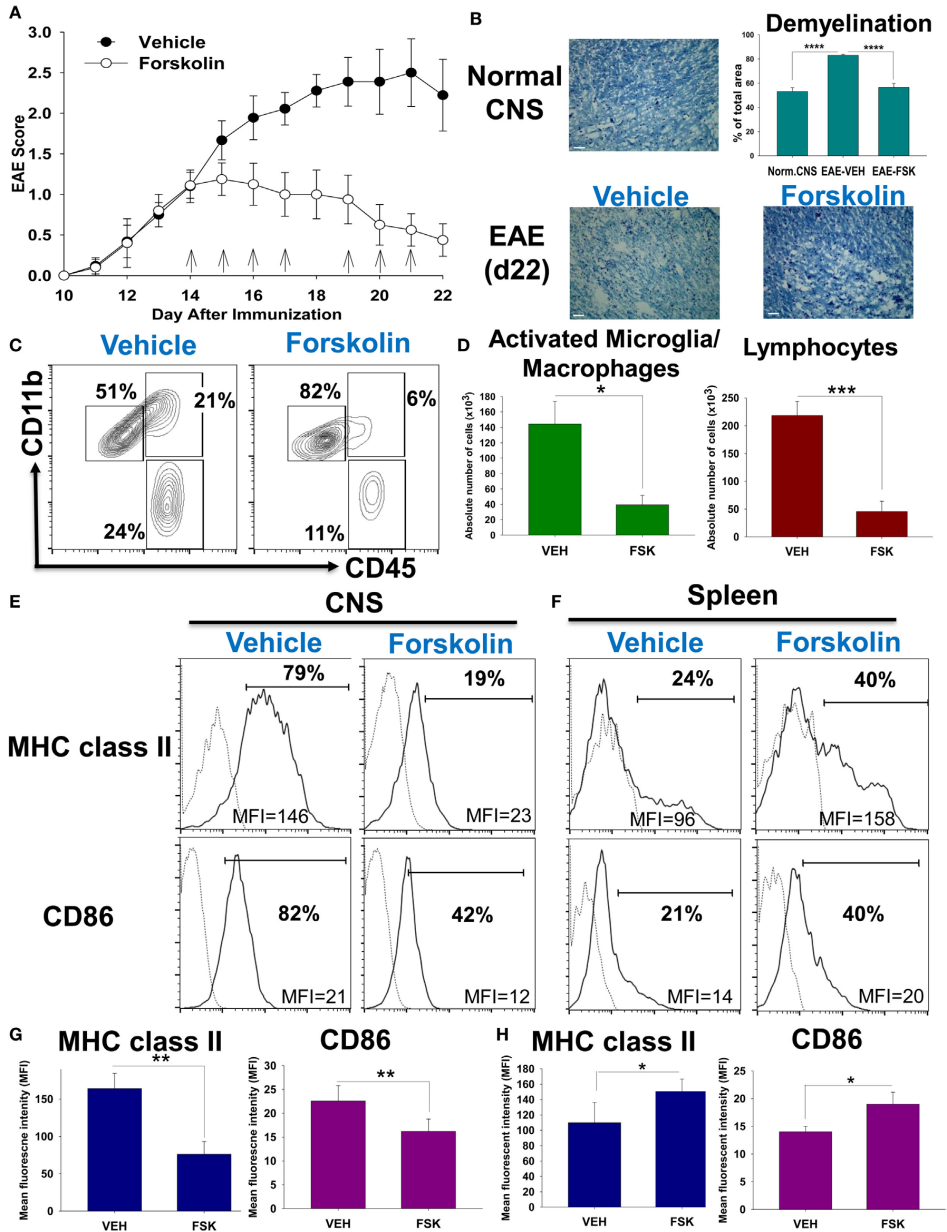
Analysis of the role of Forskolin in the modulation of neuroinflammation and expression of MHC class II and CD86 in the central nervous system (CNS) and periphery. EAE was induced and vehicle (PBS with 1% DMSO) or Forskolin were administrated i.p. on days 14, 15, 16, 17, 19, 20, and 21 post-immunization as described in Section “Materials and Methods.” On day 22, mice were perfused with cold PBS and brains, spinal cords, and spleen were isolated for histology **(B)** and/or flow cytometry analysis **(C–H)**. **(A)** EAE clinical course (mean ± SE) of total 11–12 individual mice for each group is shown. Injections of vehicle or Forskolin are indicated by arrows. **(B)** Histology analysis of extent of CNS myelination in coronal sections of lumbar area of the spinal cords of unmanipulated mice (top image) or mice with EAE on day 22 (bottom images) treated with vehicle (left image) or Forskolin (right image). Extent of myelination was assessed in lumbar area of the spinal cord using luxol fast blue (LFB) dye as described in Section “Materials and Methods” (bar: 50 μm; magnification: 400×). Quantitative analysis of percentage of myelin-free LFB-negative areas is shown on bar graph on the right. Mean ± SE of six separate images from three individual mice is shown (****p < 0.0001). **(C,D)** The flow cytometry analysis of the CNS mononuclear cells isolated from the mice with EAE on day 22 treated with vehicle or Forskolin. The mononuclear cells were isolated from the CNS of mice with EAE, stained for CD11b and CD45, and analyzed by flow cytometry as described in Section “Materials and Methods.” The percentages of populations of CD11b^+^CD45^low^ microglia (left gates), CD11b^+^CD45^hi^ macrophages (upper right gates), and CD11b^−^CD45^hi^ lymphocytes (lower right gates) are shown. The quantification of the absolute number of macrophages and lymphocytes in the CNS is shown in panel **(D)**. **(E–H)** The flow cytometry analysis of expression of M1-associated markers in macrophages in the CNS **(E,G)** or spleens **(F,H)** of mice with EAE on day 22 treated with Vehicle or Forskolin. Mononuclear cells from CNS **(E,G)** or spleens **(F,H)** were stained for CD11b, F4/80, MHC class II, and CD86 and CD11b^+^F4/80^+^ cells were analyzed for the expression of MHC class II and CD86 by four-color cytometry as described in Section “Materials and Methods.” Representative histograms for MHC class II (upper row) and CD86 (bottom row) are shown in panels **(E,F)**. A solid line indicates staining for MHC class II or CD86 and a dotted line indicates staining for isotype-matched control. A bar indicates percentage of MHC class II or CD86-positive cells. Mean fluorescence intensity (MFI) of expression of MHC class II or CD86 is shown at the bottom of each histogram. Quantitative analyses of MHC class II and CD86 expression in macrophages in the CNS and spleens are shown in panels **(G,H)**, respectively. In panels **(D,G,H)**, mean ± SE of five individual animals is shown (*p < 0.05; **p < 0.01; ***p < 0.001; NS, not significant).

Based on literature, we hypothesized that administration of Forskolin would lead to activation of cAMP pathway in various cell types that might be beneficial for suppression of EAE by inhibiting autoimmune CD4 T cells and/or changing balance of macrophage polarization from M1 to M2. We started administration of Forskolin on the day of onset of the disease, when encephalitogenic IFNγ and IL-17 producing Th1 and Th17 cells were primed and already migrated into CNS leading to appearance of fist clinical symptoms (Figure 1A, day 14). Administration of Forskolin substantially ameliorated the disease leading to recovery, while control vehicle-treated group had peak of the disease on days 21–22 (Figure 1A). We found that mice treated with Forskolin had very low level of demyelination in the spinal cord, as determined by LFB staining (Figure 1B). Forskolin-treated mice had substantially lower level of percentages and absolute numbers of CD11b^+^CD45^hi^ macrophages/activated microglia and CD11b^−^CD45^hi^ lymphocytes in the CNS on day 22 (Figures 1C,D). Forskolin-treated mice had also diminished level of expression of MHC class II and CD86 on CD11b^+^F4/80^+^ microglia/macrophages in the CNS (Figures 1E,G) but not on CD11b^+^F4/80^+^ macrophages in the spleen (Figures 1F,G). Thus, we found that Forskolin was effective in downmodulation of CNS autoimmune inflammation leading to decrease in expression of activation marker MHC class II and M1 marker CD86.

### Forskolin Upregulated Arg1 and Inhibited NOS2 in the CNS during EAE by Changing Balance toward M2

**Figure 2:**
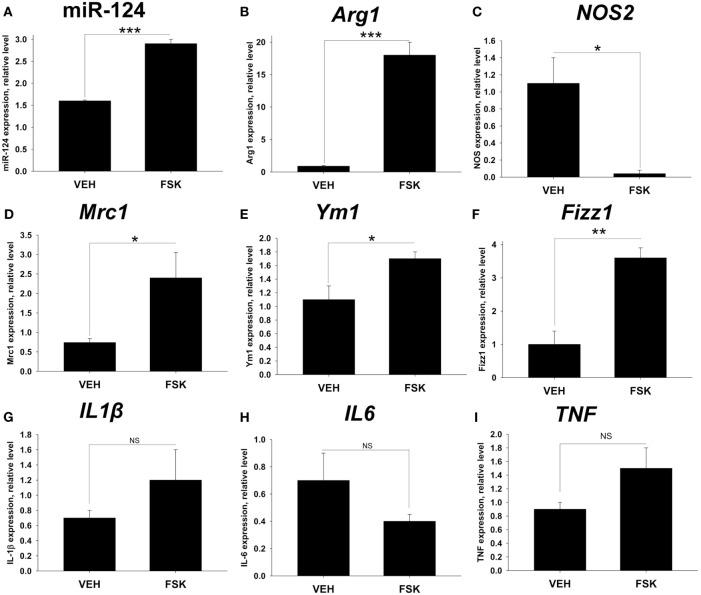
The effect of Forskolin on skewing balance toward M2 phenotype in the central nervous system during EAE. EAE was induced, and vehicle or Forskolin was administrated i.p. on days 14, 15, 16, 17, 19, 20, and 21 post-immunization similarly as for Figure 1. On day 22, mice were perfused with cold PBS, and the spinal cords were isolated for RNA isolation and analysis. Expression of M2 markers [microRNA (miR)-124 **(A)**, *Arg1* **(B)**, *Mrc1* **(D)**, *Ym1* **(E)**, and *Fizz1* **(F)**], M1 markers [*NOS2* **(C)**, *TNF* **(I)**], and general pro-inflammatory markers [IL-1β **(G)**, IL-6 **(H)**] was performed by real-time RT-PCR as described in Section “Materials and Methods.” In panels **(A–I)**, mean ± SE of triplicate is shown (*p < 0.05; **p < 0.01; ***p < 0.001; NS, not significant).

We hypothesized that downregulation of general activation marker MHC class II and M1 marker CD86 on microglia/macrophages in the CNS of Forskolin-treated mice with EAE was due to changing balance toward M2. To further verify this, we investigated the expression of additional numbers of M1 (NOS2, TNF), M2 markers (miR-124, Arg1, Mrc1, Ym-1, Fizz1), and general macrophage activation markers (IL-1β, IL-6) in the CNS of control group vs. Forskolin-treated group of mice with EAE on day 22. We found that miR-124, *Arg1, Mrc1, Ym1*, and *Fizz1* were upregulated in Forskolin-treated mice (Figures 2A,B,D–F), while *NOS2* was downregulated (Figure 2C). We did not find statistically significant difference for *IL-1β, IL6*, and *TNF* (Figures 2G–I). However, *IL-1β* and *TNF* had trend to be elevated (Figures 2G,I) and *IL-6* had a trend to be decreased (Figure 2H). Notably, we observed the dramatic 19-fold upregulation for *Arg1* (Figure 2B) and almost undetectable level of *NOS2* (Figure 2C) in the spinal cord of Forskolin-treated mice with EAE. Dramatic upregulation of *Arg1* was in excellent agreement with our *in vitro* data (Figure 2B). Thus, we found that Forskolin skewed the balance toward M2 in the CNS during EAE leading to recovery form disease.

### Forskolin Decreased Proliferation and IFNγ Production by Autoimmune CD4 T Cells in the CNS, but Not in the Periphery

**Figure 3:**
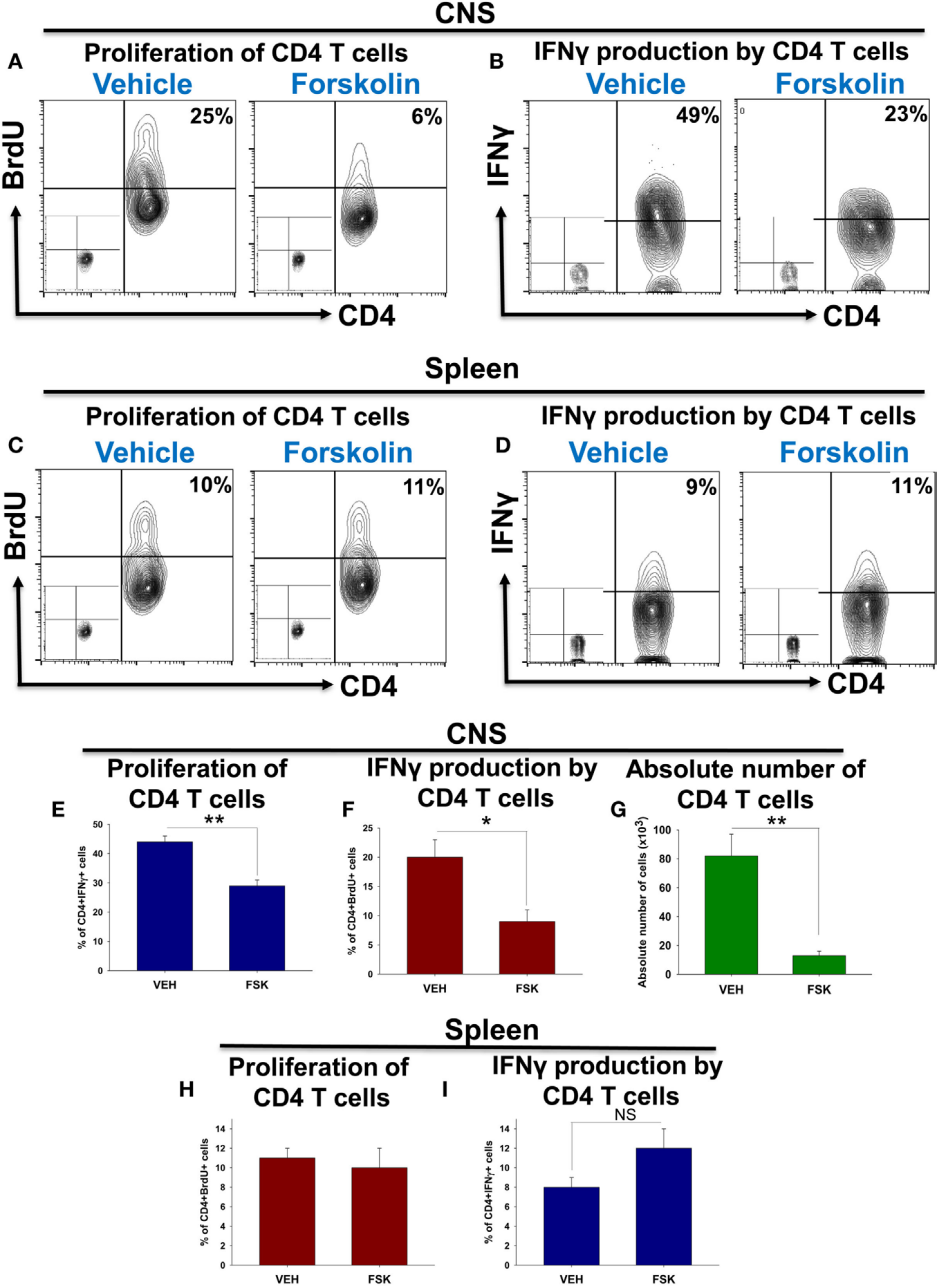
The effect of Forskolin on proliferation and IFNγ production by autoimmune CD4 T cells in the central nervous system (CNS) and periphery during EAE. EAE was induced, and vehicle or Forskolin was administrated i.p. on days 14, 15, 16, 17, 19, 20, and 21 post-immunization similarly as for Figure 1. On day 21, BrdU was injected i.p. as described in Section “Materials and Methods.” On day 22, mononuclear cells were isolated from CNS or periphery (spleen) as for Figure 1 and CD4 T cells were analyzed for the expression of surface markers, BrdU incorporation and intracellular IFNγ expression as described in Section “Materials and Methods.” **(A,C,E,H)** To measure CD4 T cell proliferation, BrdU was injected i.p. 14 h before analysis and mononuclear cells were isolated from CNS **(A,E)** or spleen **(C,H)**. The cells were stained for CD4 (*x*-axis) and BrdU (*y*-axis) and analyzed with two-color flow cytometry. BrdU incorporation by CD4^+^ gated cells is shown on representative contour-plots **(A,C)**, and statistics is shown in panels **(E,H)**. Percentages of BrdU-positive CD4^+^ gated cells are shown on the upper left quadrant of each contour-plot. Negative controls for BrdU staining are shown at left bottom quadrant of each contour-plot. **(B,D,F,I)** To measure production of IFNγ by CD4 T cells, mononuclear cells were isolated from CNS **(B,F)** or spleen **(D,I)** and stimulated with PMA/ionomycin in the presence of *GolgiStop* for 4 h as described in Section “Materials and Methods.” Then, the cells were stained for CD4 (*x*-axis) and IFNγ (*y*-axis) and analyzed with two-color flow cytometry. IFNγ production by CD4^+^ gated cells is shown on representative contour-plots **(B,D)**, and statistics is shown in panels **(F,I)**. Percentages of IFNγ-positive CD4^+^ gated cells are shown on the upper left quadrant of each contour-plot. Negative controls for IFNγ staining are shown at left bottom quadrant of each contour-plot. **(G)** Mononuclear cells were stained with CD4 and TCRβ and absolute number of CD4^+^TCRβ^+^ cells was quantified as for Figure 8. In panels **(E–I)**, mean ± SE of three to five individual mice is shown (*p < 0.05; **p < 0.01; NS, not significant).

Since it was reported that Forskolin could decrease proliferation of T cells, we checked the level of proliferation of CD4 T cells in the CNS and periphery (spleen) during EAE on day 22. We found that that proliferation of CD4 T cells was decreased twofold from 20 ± 3 to 9 ± 2% in the CNS of Forskolin-treated mice (Figures 3A,E). The level of production of IFNγ by CD4 T cells was also decreased in the CNS 1.5-fold, indicating decreased level of activation and differentiation of pathogenic Th1 cells (Figures 3B,F). This was consisted with 6.3-fold decrease in absolute number of infiltrating CD4 T cells in CNS of mice with EAE on day 22 (Figure 3G). At the same time, Forskolin did not influence the level of proliferation of CD4 T cells in the spleen (Figures 3C,H). It was even trend for increase in IFNγ production in the spleen of Forskolin-treated mice with EAE on day 22; however, it was not statistically significant (Figures 3D,I). We also found that during peak of EAE (day 22) mRNA levels for both Th1 and Th17 cytokines IFNγ and IL-17A were decreased ~8-fold in the spinal cords of Forskolin-treated mice when compared with vehicle-treated group indicating decrease in activity of both Th1 and Th17 cells (data not shown). Thus, these data demonstrate that administration of Forskolin decreased proliferation and pro-inflammatory cytokine production by pathogenic CD4 T cells in the CNS at the site of inflammation, but not in the periphery.

### Forskolin Did Not Have Direct Effect on Proliferation of MOG-Specific Autoimmune CD4 T Cells

**Figure 4:**
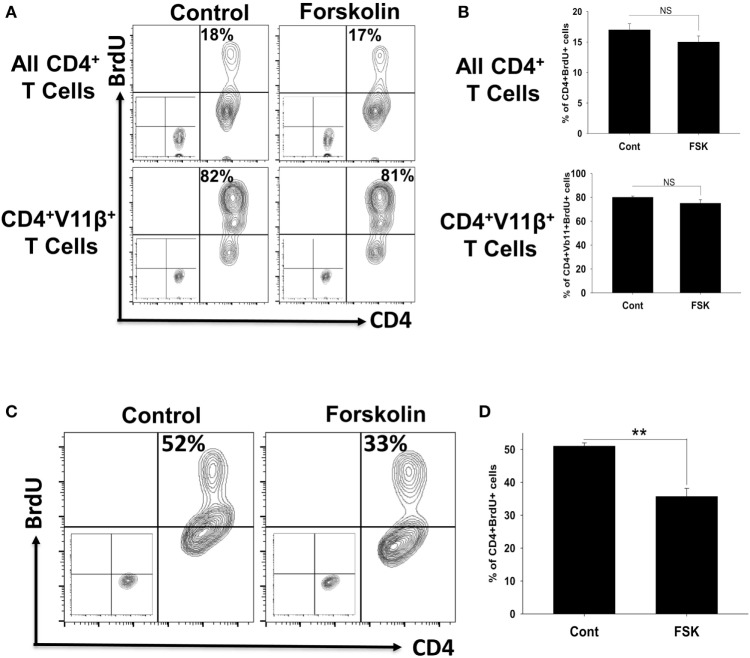
Analysis of direct effect of Forskolin on the proliferation of myelin oligodendrocyte glycoprotein (MOG)-specific autoimmune CD4 T cells and polyclonally stimulated CD4 T cells with anti-CD3/CD28. **(A,B)** The influence of Forskolin on the proliferation of MOG-specific autoimmune CD4 T cells. Mice were immunized with MOG_35–55_, and on day 7 splenocytes and the cells from draining lymph nodes were isolated. CD4 T cells were purified using magnetic beads and cultured with irradiated splenocytes for 72 h without Forskolin for the control sample or with Forskolin (FSK) as described in Section “Materials and Methods.” To measure CD4 T cell proliferation, BrdU was added 14 h before analysis. The cells were stained for CD4 (*x*-axis), TCRVβ11, and BrdU (*y*-axis), and analyzed with three-color flow cytometry. Total CD4^+^ gated cells are shown on upper contour-plots, while CD4^+^TCRVβ11^+^ gated cells are shown in bottom contour-plots in panel **(A)**. Percentages of BrdU-positive CD4^+^ gated (top contour-plots) or CD4^+^TCRVβ11^+^ gated (bottom contour-plots) CD4 T cells are shown on the upper left quadrant of each contour-plot. Negative controls for BrdU staining are shown at left bottom quadrant of each contour-plot. Mean ± SE of four culture wells is shown in panel **(B)** (NS, not significant). **(C,D)** The influence of Forskolin on the proliferation of polyclonally stimulated CD4 T cells with anti-CD3/CD28. Percentages of BrdU-positive CD4^+^ T cells are show on the upper left quadrant of each contour-plot **(C)**. Negative controls for BrdU staining are shown in a left bottom quadrant of each contour-plot. Mean ± SE of four culture wells is shown in panel **(B)** (***p < 0.001).

Although we found dramatic effect of Forskolin on EAE disease course, macrophage polarization, and proliferation of CD4 T cells in the CNS, effect of Forskolin on MOG-specific CD4 T cells could be secondary due to skewing macrophages toward M2, which poorly stimulate proliferation and differentiation of Th1 cells. It was reported that Forskolin directly inhibit proliferation of naïve T cells and T cell lines stimulated with anti-CD3, but the role of Forskolin on proliferation of effector T cells remained controversial (23). In our experiments, we started treatment after disease onset, when encephalitogenic T cells where already primed and become effector CD4 T cells. These effector CD4 T cells were not inhibited by Forskolin in the spleen and had level of proliferation and IFNγ production similar to control group. This may indicated that Forskolin had no direct effect on autoimmune T cells in the periphery. To address this, we further investigated whether Forskolin directly affect proliferation of primed MOG-specific CD4 T cells *in vitro* and found that Forskolin had very slight trend to decrease proliferation of CD4 T cells form 17 ± 1 to 15 ± 1% of BrdU-positive cells; however, this difference was statistically insignificant (Figures 4A,B; upper figures). When we compared extent of proliferation of MOG-specific enriched population of CD4^+^Vβ11^+^ T cells, the level of proliferation was also very similar for control or Forskolin-treated cells comprising 70–80% of CD4^+^Vβ11^+^BrdU^+^ cells (Figures 4A,B; bottom figures). Interestingly when we used anti-CD3/CD28 mAbs to elicit polyclonal stimulation in the same experiment instead of stimulation with MOG antigen, we found that Forskolin induced statistically significant 40% decrease in CD4 T cell proliferation (Figures 4C,D), as was reported earlier (2, 23). These data demonstrated that in contrast to polyclonally stimulated CD4 T cells, primed MOG-specific CD4 T cells were not susceptible to direct action of Forskolin. Thus, we demonstrate that Forskolin did not have significant direct effect on proliferation of autoimmune MOG-specific CD4 T cells in our model.

### Forskolin Synergize IL-4 in Upregulation of miR-124 and Arg1 Even in the Presence of IFNγ

**Figure 5:**
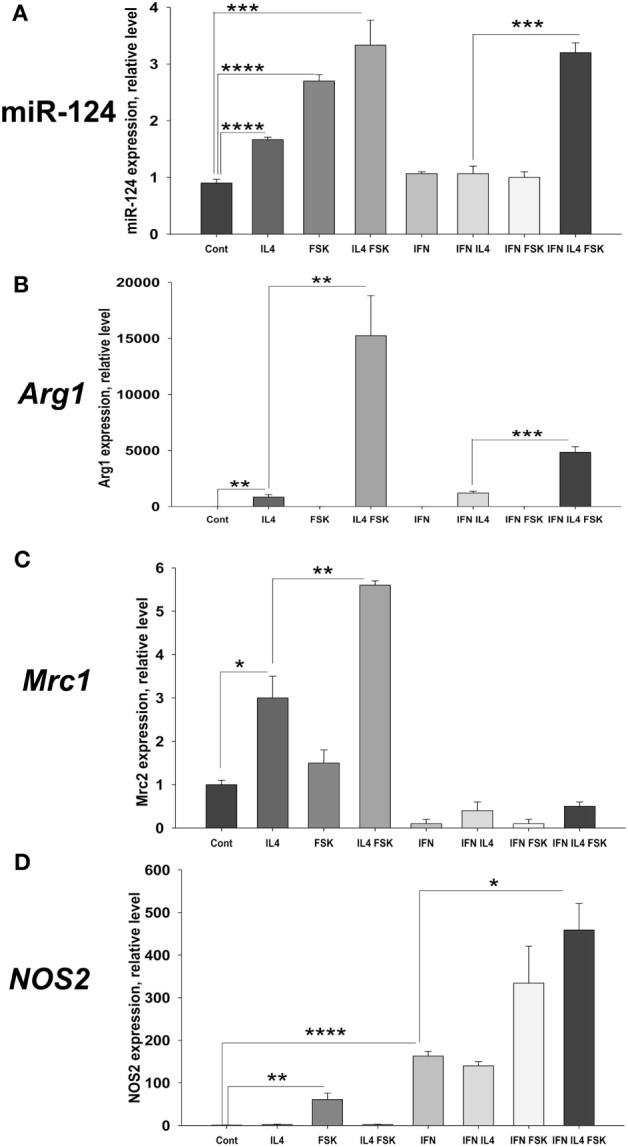
Effect of Forskolin on the expression microRNA (miR)-124, arginase (Arg)1, Mrc1, and nitric oxide synthetase (NOS)2 in bone marrow (BM)-derived macrophages at the baseline state (Control), M2 polarizing conditions (IL-4), M1 condition (IFNγ), or mixed inflammatory conditions (IL-4 and IFNγ). BM-derived macrophages were grown for 5 days in the presence of M-CSF as described in Section “Materials and Methods” and were treated with Forskolin, or IL-4, or IFNγ, or Forskolin/IL-4, or Forskolin/IFNγ, or Forskolin/IL-4/IFNγ. After 24 h, the expressions of miR-124 **(A)**, *Arg1* **(B)**, *Mrc1* **(C)**, and *NOS2* **(D)** were analyzed by real-time RT-PCR as described in Section “Materials and Methods.” Mean ± SE of four to eight culture wells is shown (*p < 0.05; **p < 0.01; ***p < 0.001; ****p < 0.0001).

Since it was recently demonstrated that cAMP contributed to M2 polarization (24, 25), we hypothesized that Forskolin could enhance IL-4 in M2 polarization. We analyzed the expression of M2-associated molecules miR-124, Arg1, Mrc1, Ym1, Fizz1, and M1-associated NOS2 in the presence of Forskolin in M0 (Control), M2 (IL-4), M1 (IFNγ), and mixed M1/M2 (IL-4 and IFNγ) conditions. Experiments demonstrated that combination of Forskolin with IL-4 resulted in 3- to 5-fold upregulation of miR-124 with average 3.3-fold (Figure 5A, IL4 FSK). However, more strikingly we found 3.4-fold upregulation of miR-124 when Forskolin was used together with IL-4 and IFNγ (Figure 5A, IL-4 IFN FSK). At the same time, IL-4 and IFNγ failed to upregulate miR-124. This indicates that Forskolin overcome inhibitory action of IFNγ in IL-4-induced miR-124 expression, when IL-4 was used together with IFNγ.

In addition to miR-124, Forskolin substantially enhanced IL-4 in induction of *Arg1* in M2 macrophages (Figure 5B). Forskolin synergized expression of *Arg1* by 18-fold when compared with the action of IL-4 alone (Figure 5B; IL4 and IL4 FSK). Forskolin upregulated *Arg1* by fivefold in mixed inflammatory conditions in the presence of IL-4 and IFNγ (Figure 5B; IFN IL4 and IFN IL4 FSK). This drug also enhanced expression of M2 marker *Mrc1*; however, it failed to induce it in mixed inflammatory conditions (Figure 5C). Finally, Forskolin did not have substantial effect on IL-4-induced expression of two other tested M2 markers *Ym1* and *Fizz1* (data not shown).

When we compared expression of M1 markers in IL-4- and IFNγ-activated macrophages, we found that Forskolin induced low level of *NOS2* expression by itself and enhanced induction of *NOS2* by IFNγ (Figure 5D). Although *Arg1* and *NOS2* were shown to be antagonistic markers, Forskolin induced both in mixed inflammatory conditions, but the level of *Arg1* was more than 10-fold higher than *NOS2* (Figures 5B,D, IFN IL4 FSK). Finally, Forskolin did not affect the expression of other M1 markers CD86 and *TNF* (data not shown).

Taken together, these data indicate that Forskolin synergize action of IL-4 in induction of miR-124 and *Arg1* in the M2-skewing condition in the presence of IL-4 and mixed inflammatory conditions (IL-4 with IFNγ) changing balance toward M2.

### Forskolin Activate ERK and Synergize Activation of ERK Pathway by IL-4

We further investigated mechanism by which Forskolin induce expression of M2 markers. It was reported that CEBPβ could become phosphorylated by ERK1/2 in response to IFNγ in mouse macrophage cell line that play an important role in IFNγ-inducible gene expression such as *NOS2* (26, 27). On the other hand, CREB–CEBPβ axis was shown to become activated in LPS-treated macrophages resulting in expression of number M2 genes including *Arg1* and *Mrc1* while M1 genes remained unaffected (28). Thus, the roles of ERK–CEBPβ and CREB–CEBPβ axes in M1 vs. M2 remained unclear. We tested whether IL-4 and/or Forskolin affect activation CREB, ERK, and CEBPβ by inducing their upregulation and/or phosphorylation.

**Figure 6:**
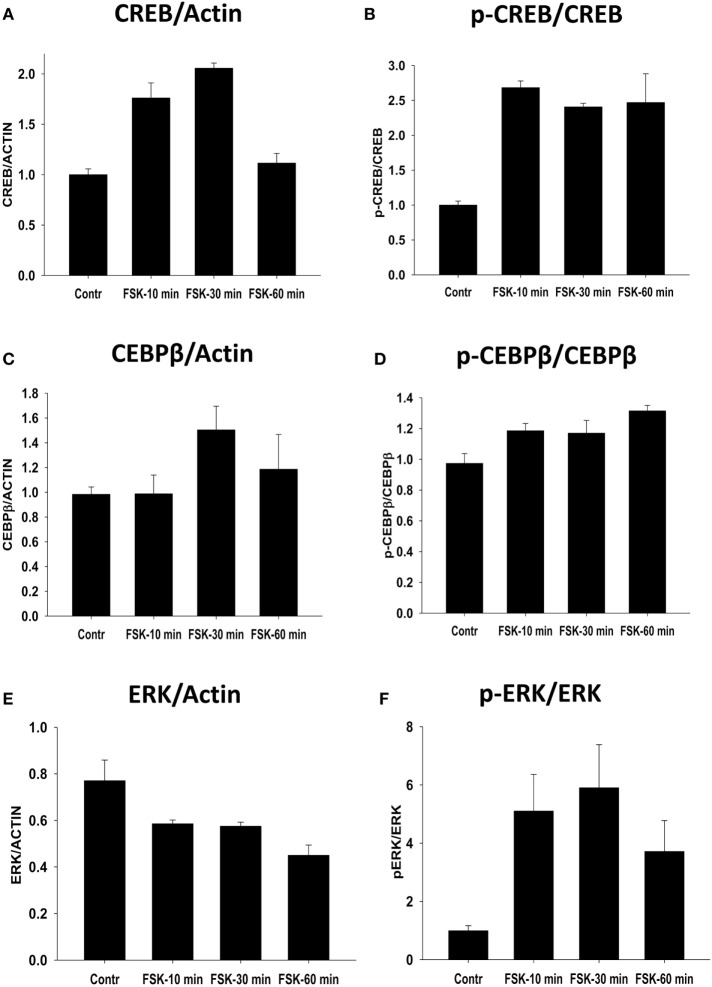
Effect of Forskolin on early activation of downstream signaling pathways CREB, CEBPβ, and ERK1/2. Bone marrow-derived macrophages were grown for 5 days in the presence of M-CSF and analyzed as untreated (Control), or were treated with Forskolin (FSK) for 10, 20, or 30 min. Expressions of CREB, CEBPβ, and ERK1/2 and their phosphorylated forms (p-CREB, p-CEBPβ, and p-ERK) were analyzed by Western blot as described in Section “Materials and Methods.” β-Actin was used as a loading control. **(A,C,E)** Quantitative analysis of relative expression levels of CREB, CEBPβ, and ERK normalized to β-actin is shown. Representative image is shown in Figure S1 in Supplementary Material. **(B,D,F)** Quantitative analysis of relative expression levels of p-CREB, p-CEBPβ, and p-ERK normalized to total CREB, CEBPβ, and ERK1/2, respectively, is shown. Representative image is shown in Figure S1 in Supplementary Material. In panels **(A–D)**, mean ± SE of three separate experiments is shown.

First, we investigated early kinetics of expression level and extent of phosphorylation of CREB, ERK, and CEBPβ after 10, 30, and 60 min of incubation with Forskolin. We found that CREB upregulation (Figure 6A; Figure S1 in Supplementary Material) and phosphorylation (Figure 6B; Figure S1 in Supplementary Material) was detected 10–60 min after incubation with Forskolin. We found very modest upregulation of CEBPβ (Figure 6C; Figure S1 in Supplementary Material) and only slight increase in CEBPβ phosphorylation (Figure 6D; Figure S1 in Supplementary Material) after 30–60 min of incubation with Forskolin. Forskolin induced subtle downregulation (Figure 6E; Figure S1 in Supplementary Material) and substantial increase in the level of phosphorylation of ERK1/2, which was detectable after 10–60 min of incubation with Forskolin (Figure 6F; Figure S1 in Supplementary Material).

**Figure 7:**
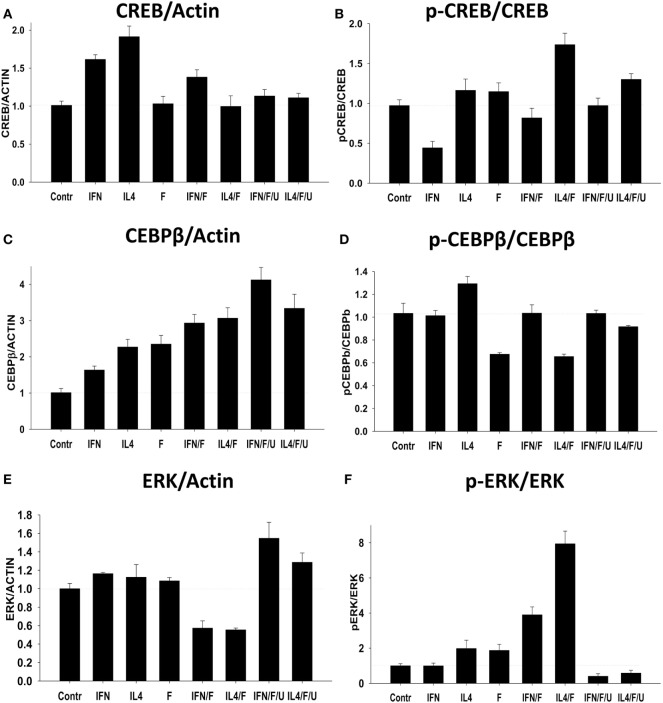
Effect of Forskolin on activation of downstream signaling pathways CREB, CEBPβ, and ERK1/2 in M0 macrophages (Control), or during M2 (IL-4) vs. M1 (IFNγ) polarizing conditions. Bone marrow-derived macrophages were grown for 5 days in the presence of M-CSF and were treated with Forskolin, or IL4, or IFNγ, or Forskolin/IL4, or Forskolin/IFNγ, or Forskolin/IL4/IFNγ for 24 h as in Figure 1. ERK1/2 inhibitor U0126 was used to block ERK1 and ERK2 phosphorylation (see Materials and Methods). Expressions of CREB, CEBPβ, and ERK1/2 and their phosphorylated forms (p-CREB, p-CEBPβ, and p-ERK1/2) were analyzed by Western blot as described in Section “Materials and Methods.” β-Actin was used as a loading control. **(A,C,E)** Quantitative analysis of relative expression levels of CREB, CEBPβ, and ERK normalized to β-actin is shown. Representative image is shown in Figure S1 in Supplementary Material. **(B,D,F)** Quantitative analysis of relative expression levels of p-CREB, p-CEBPβ, and p-ERK normalized to total CREB, CEBPβ, and ERK1/2, respectively, is shown. Representative image is shown in Figure S1 in Supplementary Material. In panels **(A–D)**, mean ± SE of three separate experiments is shown. Abbreviations: IFN, IFNγ; IL4, IL-4; F, Forskolin; U, U0126.

Second, we investigated long-term effect of Forskolin and/or M1 (IFNγ) and M2 (IL-4) activating stimuli on expression and extent of phosphorylation of CREB, ERK, and CEBPβ after 24 h of incubation with Forskolin. We found that CREB was upregulated by IFNγ and IL-4 (Figure 7A; Figure S1 in Supplementary Material), while the highest level of CREB phosphorylation was observed in macrophages treated with IL-4 together with Forskolin (Figure 7B, *IL4/F*; Figure S1 in Supplementary Material), which was blocked by ERK inhibitor U0126 (Figure 7B, *IL4/F/U*; Figure S1 in Supplementary Material). Interestingly, we found that IL-4 caused the highest level of CREB expression at 24 h time-point indicating important role of cAMP in conventional IL-4-polarized macrophages (Figure 7A). Most of activating stimuli (IFNγ alone, IL-4 alone, or IL-4 with Forskolin, or IFNγ with Forskolin) upregulated CEBPβ with little difference in extent of phosphorylation of CEBPβ (Figures 7C,D; Figure S1 in Supplementary Material). ERK inhibitor did not downregulate expression of CEBPβ (Figure 7C). ERK expression was not influenced by most of activating stimuli while IFN/Forskolin and IL-4/Forskolin even decreased it (Figure 7E; Figure S1 in Supplementary Material). However, phosphorylation of ERK1/2 was slightly enhanced by IL-4 or Forskolin, but the highest level of ERK phosphorylation was observed when IL-4 was added together with Forskolin (Figure 7E; Figure S1 in Supplementary Material). In contrast, IFNγ alone did cause substantial phosphorylation of ERK, but Forskolin with IFNγ substantially enhanced ERK1/2 phosphorylation. The ERK inhibitor U0126 blocked phosphorylation of ERK1/2 caused by IL-4 together with Forskolin, or IFNγ with Forskolin (Figure 7E). Thus, we concluded that Forskolin enhanced action of IL-4 in phosphorylation of ERK1/2 and CREB, but ERK pathway did not play a substantial role in CEBPβ phosphorylation.

### ERK Inhibitors Block Upregulation of Arg1, Mrc1, and miR-124 Induced by Forskolin with IL-4

We found that Forskolin activated ERK1/2 pathway and stimulated expression of M2 markers. We hypothesized that Forskolin induced expression of M2 markers and miR-124 *via* activation ERK1/2. However, currently, it is not clear whether ERK1/2 promote M2 polarization or not. It was reported that ERK1/2 activation contributed to both M1 and M2 phenotypes (26, 29–32). The role of ERK was shown to be important for Th2-mediated allergic lung inflammation (33), while the role of miR-124 was also shown in our studies to be important for EAE and allergic lung inflammation (10, 17). Therefore, we further investigated the role of ERK pathway in upregulation of miR-124, *Arg1, Mrc1*, and *NOS2* induced by IL-4 together with Forskolin. We found that in the presence of ERK inhibitor U0126 the expression of miR-124 fell below control level (Table 1, miR-124). In addition to U0126, we used another ERK inhibitor P184352 to confirm inhibitory effect of U0126. Both ERK inhibitors U0126 and P184352 inhibited Arg1 expression by 58 and 98%, respectively (Table 1, Arg1). Both inhibitors U0126 and P184352 increased expression of NOS2 by fourfold and sevenfold, respectively (Table 1, NOS2). At the same time, U0126 decreased expression of *Mrc1* by 50%, upregulated expression of *Ym1* by 25%, and had no effect on *Fizz1* expression (Table 1). Thus, we found that ERK pathway substantially contributed to upregulation of miR-124 and *Arg1* and downregulation of *NOS2* caused by the simultaneous action of IL-4 and Forskolin. At the same time, we found that Forskolin enhanced IFNγ to induce expression of *NOS2* (Figure 5D, NOS2). We investigated whether ERK pathway contributed to this process as it was described earlier for RAW 264.7 cell line (26). We did find that ERK pathway partially contributed to IFNγ-inducible *NOS2* expression since U0126 decreased expression of *NOS2* by 21% (Table 2, NOS2). However, inhibition of ERK pathway resulted in twofold increase in *TNF* expression (Table 2), indicating that ERK partially contributed to IFNγ-inducible NOS but not other M1 marker TNF. Thus, ERK pathway contributed to upregulation of M2 markers miR-124 and *Arg1* (Table 1) and partially *NOS2* and downmodulation of *TNF* (Table 2). These data demonstrated importance of ERK pathway in the expression of M2 markers miR-124 and Arg1.

**Table 1:** Effect of ERK inhibitors U0126 and PD184352 on IL-4/Forskolin-induced expression of microRNA (miR)-124, *Arg1, NOS2, Mrc1, Ym1*, and *Fizz1*.^a^

Marker	Untreated	IL-4 FSK	IL-4 FKS U0126	IL-4 FSK PD184352
*miR-124*	1.0 ± 0.1	2.6 ± 0.2	0.8 ± 0.05^b^	ND
*Arg1*	1.0 ± 0.2	8,206 ± 442	3,463 ± 74^b^	180 ± 13^c^
*NOS2*	1.0 ± 0.2	9 ± 2	38 ± 7^d^	65 ± 11^d^
*Mrc1*	1.0 ± 0.1	5.6 ± 0.1	2.6 ± 0.1^c^	ND
*Ym1*	1.0 ± 0.2	15 ± 1	20 ± 1^d^	ND
*Fizz1*	1.0 ± 0.1	2.7 ± 0.1	2.8 ± 0.2	ND

*^a^Bone marrow (BM)-derived macrophages were grown for 5 days in the presence of M-CSF and were analyzed as untreated (control) or treated with IL-4 and Forskolin without or with ERK inhibitors U0126 or PD184352 for 24 h as for Figure 5. Relative levels compared to control are shown for all samples. Mean ± SE of three to six separate wells are shown. These data are representative of three separate experiments*.

*^b^*p* < 0.001 when compared to IL-4/Forskolin (IL-4 FSK) treated cells*.

*^c^*p* < 0.0001 when compared to IL-4/Forskolin (IL-4 FSK) treated cells*.

*^d^*p* < 0.01 when compared to IL-4/Forskolin (IL-4 FSK) treated cells*.

*ND, not determined*.

**Table 2:** Effect of ERK inhibitor U0126 on IFNγ/Forskolin-induced expression of *NOS2* and *TNF*.^a^

Marker	Untreated	IFNγ FKS	IFNγ FSK U0126
*NOS2*	1.0 ± 0.2	431 ± 20	355 ± 8^b^
*TNF*	1.0 ± 0.1	1.2 ± 0.1^d^	2 ± 0.2^c^

*^a^Bone marrow (BM)-derived macrophages were grown for 5 days in the presence of M-CSF and were analyzed as untreated (control) or treated with IFNγ and Forskolin without or with ERK inhibitor U0126 for 24 h as for Figure 5. Relative levels compared to control are shown for all samples. Mean ± SE of three to six separate wells are shown. These data are representative of three separate experiments*.

*^b^*p* < 0.01 when compared to IFNγ/Forskolin (IFNγ FSK) treated cells*.

*^c^*p* < 0.05 when compared to IFNγ/Forskolin (IFNγ FSK) treated cells*.

### ERK Inhibitor Block Upregulation of Arg1, Mrc1, and Cyclin D2 and Upregulate NOS2 in IL-4 Activated BM-Derived Macrophages

**Figure 8:**
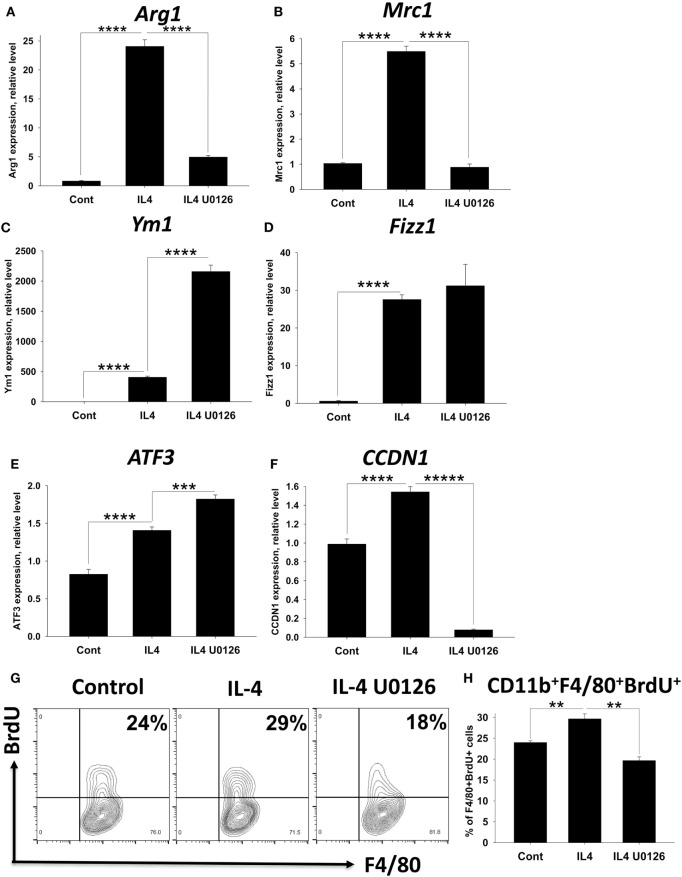
Analysis of the role of ERK pathway in upregulation of M2 markers and proliferative capacity of bone marrow (BM)-derived macrophages polarized with IL-4. BM-derived macrophages were grown for 5 days in the presence of M-CSF and were analyzed as untreated (control) or treated with IL-4, or IL-4 with ERK inhibitor U0126 for 24 h as for Figure 2. **(A–F)** Analysis of M2 marker expression. To assess expression of M2-associated and proliferation markers, RNA was isolated and the expression of *Arg1, Mrc1, Ym1, Fizz1, ATF3*, and *CCDN1* (Cyclin D1) was analyzed by real-time RT-PCR as described in Section “Materials and Methods.” **(G,H)** Analysis of macrophage expansion. To assess macrophages proliferation, BrdU was added 14–16 h before analysis to cell cultures, after which the cells were stained for surface markers CD11b and F4/80 and intracellular BrdU and analyzed by three-color flow cytometry as described in Section “Materials and Methods.” Representative contour-plots for CD11b^+^F4/80^+^ gated cells are shown in panel **(G)**. Expression of F4/80 is shown on *x*-axis and expression of BrdU is shown on *y*-axis. Percentages of BrdU-positive cells are shown on upper right quadrants of each contour-plot. Quantitative analysis for mean ± SE of percentages of CD11b^+^F4/80^+^BrdU^+^ cells is shown in panel **(H)**. In panels **(A–F,H)**, mean ± SE of three to five culture wells is shown (**p < 0.01; ***p < 0.001; ****p < 0.0001; *****p < 0.00001).

Since ERK inhibitors inhibited IL-4/Forskolin-induced expression of miR-124 and *Arg1* (Table 1), we hypothesized that ERK pathway is important for upregulation of M2 markers in conventional M2 macrophages polarized with IL-4 without Forskolin. Our hypothesis was also based on the finding that IL-4 alone but not IFNγ alone caused ERK1/2 phosphorylation (Figure 7). It was suggested that ERK contribute to M2 phenotype for tumor-infiltrating macrophages (TIMs) (29, 30), but the role of ERK has not be proven to be essential for IL-4-activated macrophages as a general concept. We further compared expression of several M2 markers in IL-4-polarized BM-derived macrophages with and without U0126 inhibitor. We found that ERK pathway was critical for IL-4-induced upregulation of *Arg1, Mrc1* (Figures 8A,B), but not *Ym1* and *Fizz1* (Figures 8C,D). Moreover, *Ym1* was further upregulated by ERK inhibitor U0126 (Figure 8D). We also investigated the level of expression of member of CREB/ATF family ATF3 as a transcriptional factor that is regulated by cAMP and found that ATF3 was upregulated by IL-4, suggesting possible involvement of this transcription factor in M2 polarization (Figure 8E). ERK inhibitor U0126 even further upregulated ATF3 in IL-4-treated macrophages (Figure 8E) demonstrating that other pathways and/or co-factors were responsible for the upregulation of ATF3. Thus, we confirmed ERK pathway was important for the upregulation of *Arg1* and *Mrc1* by IL-4. We also found that in addition to CREB, ATF3 might be also involved in M2 polarization process but an expression of this factor was ERK independent.

It was previously reported that M2 macrophages have proliferative capacity *in vivo* upon systemic administration of IL-4 (34). Since reported proliferation of tissue-resident macrophages in peritoneal cavity (35) and microglia in the CNS during EAE (14) could be connected with activation of ERK pathway, we investigated the level for the expression of Cyclin D (CCDN1). CCDN1 plays a central role in the regulation of cell division and it is required (but not sufficient without other co-factors such as CDK4 and CDK6) for the progression from G1 to S phase of cell cycle (36). We found that in BM-derived macrophages *CCDN1* was expressed at the baseline level, which was not surprising since these cells were expanded in culture in the presence of M-CSF. However, IL-4 further upregulated mRNA for Cyclin D1 (Figure 8F, IL4) and expression of *CCDN1* was completely blocked by ERK inhibitor (Figure 8F, IL4 U0126). This indicates that both M-CSF and IL-4 contributed to ERK-dependent expression of *CCDN1* in BM-derived macrophages. We confirmed reported study that M-CSF induced ERK phosphorylation (37) in BM-derived macrophages (data not shown) suggesting that both IL-4 and M-CSF could contribute to the ERK-dependent expression of *CCDN1*. Despite notable upregulation of *CCDN1* expression by IL-4, we did not find very dramatic difference in the proliferation of IL-4-treated macrophages *in vitro* probably due to baseline level of M-CSF-driven proliferation of these cells (Figures 8G,H). However, ERK inhibitor U0126 did decrease proliferation of IL-4-treated BM-derived macrophages by 60%, which was consistent with reduced *CCDN1* expression in the presence of ERK inhibitor (Figures 8G,H). Thus, we found that ERK pathway is critical for the expression of *Arg1, Mrc1*, and *CCDN1* and downmodulation of *NOS2*.

### ERK Inhibitor Upregulate Expression of M1 Marker CD86 in IL-4-Activated Macrophages and NOS2 in IFNγ-Activated Macrophages

**Figure 9:**
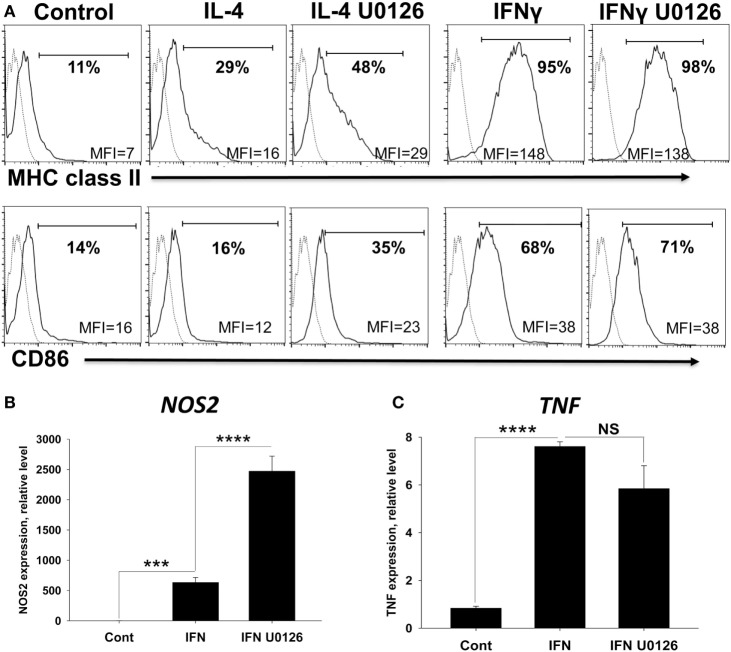
Analysis of the role of ERK pathway in upregulation of M1 markers in bone marrow (BM)-derived macrophages polarized with IFNγ. BM-derived macrophages were grown for 5 days in the presence of M-CSF and were analyzed as untreated (control) or treated with IFNγ, or IFNγ with ERK inhibitor U0126 as described in Section “Materials and Methods.” **(A)** Analysis of expression of surface markers MHC class II and CD86 on BM-derived macrophages. To assess expression of macrophage activation marker MHC class II and M1 marker CD86, macrophages were stained for CD11b and F4/80, MHC class II and CD86. CD11b^+^F4/80^+^ gated cells were analyzed for the expression of MHC class II and CD86 by four-color flow cytometry as described in Section “Materials and Methods.” A representative histogram for the expression of MHC class II (upper row) and CD86 (bottom row) of CD11b^+^F4/80^+^ gated cells is shown. The solid line indicated staining for MHC class II or CD86, and the dotted line indicated staining for isotype-matched control. The bar shows percentage of MHC class II or CD86-positive cells. The mean fluorescence intensity (MFI) of expression of MHC class II or CD86 is shown at the bottom of each histogram. **(B,C)** Analysis of expression of nitric oxide synthetase (NOS)2 and TNF M1 markers. To assess expression of two other M1-associated markers *NOS2* and *TNF*, RNA was isolated and expressions of *NOS2* and *TNF* were analyzed by real-time RT-PCR as described in Section “Materials and Methods.” In panels **(B,C)**, mean ± SE of three to five culture wells is shown (***p < 0.001; ****p < 0.0001).

We further investigated and found that ERK inhibitor U0126 upregulated expression of general activation marker MHC class II and M1 marker CD86 in IL-4-treated BM-derived macrophages, but not in IFNγ-treated macrophages (Figure 9A; Table 3). In addition, ERK inhibitor further upregulated expression of *NOS2* (Figure 9B), but not *TNF* (Figure 9C). We observed a trend of ERK inhibitor upregulating MHC class II in IFNγ-treated macrophages (Figure 9A), but this trend not statistically significant (Table 3). Thus, we found that ERK pathway was antagonistic for the expression of activation marker MHC class II and M1 markers CD86 and *NOS2*.

**Table 3:** Effect of ERK inhibitor U0126 on expression of cell surface markers MHC class II and CD86 in M2 (IL-4)- and M1 (IFNγ)-polarized bone marrow (BM)-derived macrophages.^a^

Marker	Untreated	IL-4	IL-4 U0126	IFNγ	IFNγ U0126
*MHC class II*	9 ± 1	15 ± 1	31 ± 1^b^	140 ± 7	129 ± 5^c^
*CD86*	17 ± 1	15 ± 1	23 ± 1^d^	38 ± 2	37 ± 1

*^a^BM-derived macrophages were grown for 5 days in the presence of M-CSF and were analyzed as untreated (control) or treated with IL-4 or IFNγ without or with ERK inhibitor U0126 for 24 h as for Figure 5. After, which macrophages were stained for CD11b, F4/80, MHC class II, and CD86. CD11b^+^F4/80^+^ gated cells were analyzed for the expression of MHC class II and CD86 by four-color flow cytometry as described in Section “Materials and Methods.” Mean ± SE of mean fluorescence intensity of MHC class II or CD86 on macrophages from four separate wells are shown. These data are representative of two separate experiments*.

*^b^*p* < 0.0001 when compared to IL-4 only treated cells (IL-4)*.

*^c^Not significant difference when compared to IFNγ only treated cells (IFNγ)*.

*^d^*p* < 0.01 when compared to IL-4 only treated cells (IL-4)*.

### ERK Inhibitor Block Upregulation of Arg1, Mrc1, and Cyclin D1 in IL-4-Activated Resident Peritoneal Macrophages

**Figure 10:**
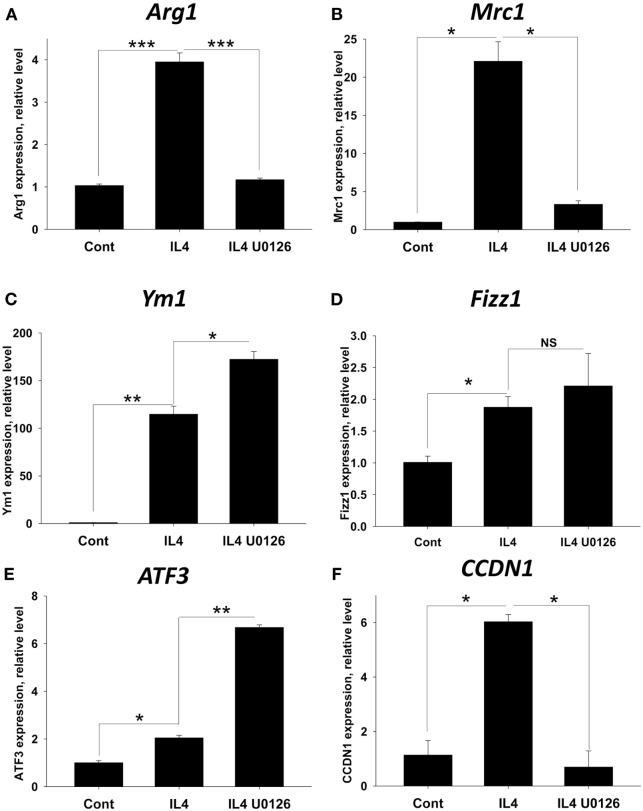
Analysis of the role of ERK pathway in the upregulation of M2-associated markers in peritoneal macrophages polarized with IL-4. Peritoneal macrophages were isolated as described in Section “Materials and Methods” and were analyzed as untreated (control) or treated with IL-4, or IL-4 with ERK inhibitor U0126 for 24 h as for Figure 8. To assess expression of M2-associated and proliferation markers, RNA was isolated and the expression of *Arg1, Mrc1, Ym1, Fizz1, ATF3*, and *CCDN1* (Cyclin D1) was analyzed by real-time RT PCR as for Figure 3. In panels **(A–F)**, mean ± SE of three to five culture wells is shown (*p < 0.05; **p < 0.01; ***p < 0.001; NS, not significant).

We confirmed our results obtained on BM-derived proliferating macrophages in non-proliferating resident peritoneal macrophages. This confirmation was also important to prove essential role of ERK in M2 polarization of various types of IL-4-stimulated macrophages. Similar to BM-derived macrophages, ERK pathway was critical for IL-4-induced upregulation of *Arg1* and *Mrc1*, but not *Ym1* or *Fizz1* (Figures 10A–D). *ATF3* was even further upregulated by ERK inhibitor U0126 when compared to BM-derived macrophages (Figure 10E). As expected, *CCDN1* was not expressed in non-proliferating peritoneal macrophages, but it was induced by IL-4 and completely blocked by ERK inhibitor (Figure 10F). We confirmed that IL-4-treated peritoneal macrophages did not proliferate *in vitro* (data not shown), indicating that other co-factors (e.g., M-CSF) and other pathways (e.g., Akt) (35) are likely to be required for their reported IL-4-driven proliferation *in vivo*. Thus, these data demonstrate the importance of ERK pathway in IL-4-induced expression of Arg1, Mrc1, and Cyclin D2 in peritoneal macrophages. This also supports our general concept that ERK is essential pathway for M2 activation.

### Forskolin Upregulate miR-124 in BM-Derived Macrophages in CREB/ATF3-Dependent Manner

We have previously found that miR-124 plays an important role in M2 macrophage polarization in the CNS and the periphery leading to suppression of EAE (10, 17). Here, we found that Forskolin affected miR-124 expression in macrophages. We hypothesized that Forskolin induced transcription of precursors of miR-124 affecting cAMP-responsive transcription factors. By performing *in silico* analysis of promoter areas of miR-124 precursor RNAs pre-miR124-1, pre-miR-124-2, and pre-miR-124-3, we found the presence of binding sites for several transcription factors from cAMP-responsive CREB/ATF family of transcription factors in pre-miR124-2 and pre-miR-124-3 promoter areas (Figure S2 in Supplementary Material). The most important factors from the list of potential regulators of miR-124 (Figure S1 in Supplementary Material) were ATF3 and CREB. CREB was upregulated by Forskolin (Figure 6A), while ATF3 was upregulated in M2 macrophages by IL-4 (Figures 8E and 10E). It was shown to be induced by IFNβ, a known FDA-approved drug (38). In addition, ATF3 was recently found to be induced in myeloid cells by dimethyl fumarate, another drug used for MS treatment (39, 40). ATF3 was also shown to promote anti-inflammatory TGFβ signaling and inhibited TNF, which is associated with the phenotype of M2 macrophages (41–43). However, when we used taurolidin, a known activator of ATF3 (44), we found only modest 1.5-fold upregulation of miR-124 (Figure S3 in Supplementary Material). It was recently shown that CREB-CREBβ axis play a major part in induction of expression of M2-associated genes *Arg1* and *Mrc1* (28). Forskolin induced early phosphorylation of CEBPβ (Figure 6B) showing activation of CREB–CEBPβ axis in macrophages by Forskolin. Thus, we found that Forskolin substantially upregulated miR-124 in macrophages acting through activation of CERB.

### REST Does Not Play a Role in Upregulation of miR-124 in Unmanipulated or Forskolin-Treated Macrophages

It was shown that miR-124 is highly expressed in neurons due to inactivation of transcriptional repressor REST (45). We also found that in contrast to neuronal cells, REST (but not the other alternative splicing isoform REST4) was highly expressed in macrophages on mRNA (Figure S4A in Supplementary Material) and protein (Figure S4B in Supplementary Material) levels. Importantly, the REST expression was not downregulated by Forskolin (Figures S4A,B in Supplementary Material). Thus, REST was not downregulated or functionally inhibited by Forskolin. Moreover, REST inhibitor X5050 failed to upregulate miR-124 in unstimulated macrophages (Figure S4C in Supplementary Material), while application of REST inhibitory RNA (NRSE) resulted in only modest 1.5-fold upregulation of miR-124 (Figure S4D in Supplementary Material). Thus, we demonstrated that Forskolin upregulated miR-124 in macrophages in REST-independent manner.

## Discussion

**Figure 11:**
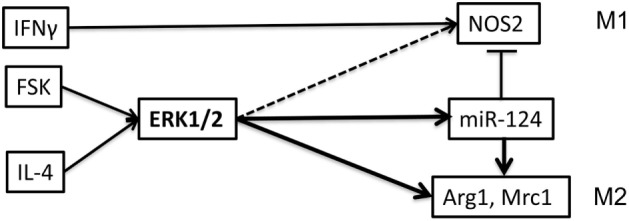
Model of changing balance toward M2 by Forskolin in mixed inflammatory conditions in the presence of IFNγ and IL4 in the central nervous system (CNS) during EAE. Our study demonstrated that IFNγ results in the upregulation of M1 marker nitric oxide synthetase (NOS)2 in an ERK-independent manner, since IFNγ alone did not result in phosphorylation of ERK. However, IFNγ and Forskolin resulted in ERK1/2 phosphorylation, contributing to NOS2 expression. IL-4 alone resulted in upregulation of M2 markers microRNA (miR)-124, arginase (Arg)1, and Mcr1 in an ERK-dependent manner. Forskolin synergized IL-4 to cause ERK phosphorylation and further upregulation of miR-124, Arg1, and Mrc1 in ERK-dependent manner. Upregulation by IL-4 and/or Forskolin miR-124 further upregulates Arg1 and downregulates NOS2, contributing to skewing macrophages toward M2 by Forskolin in the presence of both IL-4 and IFNγ. Thus, Forskolin skews balance toward M2 in mixed inflammatory conditions such as the CNS autoimmune inflammation during EAE.

This study showed that cAMP pathway is more important to modulate function of macrophages rather than T cells during EAE. We demonstrated the importance of ERK pathway for M2 polarization induced by IL-4 alone or by the synergetic action of Forskolin and IL-4 leading to the upregulation of miR-124, *Arg1*, and *Mrc1*. Forskolin also synergized action of IFNγ leading ERK phosphorylation and upregulation of NOS2; however, this effect was about 10-fold weaker when compared with stimulation of *Arg1*. In addition, we found that in contrast to Forskolin and IL-4, IFNγ alone did not induce ERK phosphorylation. We have previously found that miR-124 promote expression of *Arg1* and *Mrc1* and inhibit *NOS2* (10, 17) further contributing to M2 phenotype. Summarizing all our data, we proposed the model in which IL-4 and Forskolin activated ERK pathway that promote expression of miR-124, Arg1, and Mrc1 (Figure 11). Forskolin alone or in combination with IFNγ also promoted NOS2, but this pathway is likely antagonized by miR-124, which we found to be critical for M2 phenotype of macrophages in the CNS (10) (Figure 11). Our *in vitro* studies were further tested in *in vivo* in mouse model of autoimmune neuroinflammation demonstrating strong therapeutic effect of Forskolin and changing balance toward M2 in the CNS in mouse model of MS.

The exact role of ERK pathway in macrophage polarization remains enigmatic. It was shown that ERK pathway is required for macrophage development and M2 skewing of monocytes under the action of M-CSF during their growth and differentiation vs. M1 phenotype when monocytes are expanded in differentiated under the action of GM-CSF (30, 37). However, the role of ERK pathway in conventional M2 macrophages that were polarized with IL-4 was not investigated in details. Much less is known about the activation of ERK pathway in macrophages during EAE in mixed inflammatory conditions (IL-4 and IFNγ) and possible outcome of activation of this pathway in macrophages for the development and resolution of neuroinflammation. Our data confirmed that M-CSF-induced ERK phosphorylation, but we also demonstrated that IL-4 induced ERK1/2 phosphorylation, which was dramatically increased when IL-4 was used together with Forskolin. Moreover, we demonstrated that ERK1/2 phosphorylation was critical for the expression of miR-124, *Arg1, Mrc1*, and Cyclin D1 in M2 macrophages that were polarized by IL-4 with or without Forskolin. In addition to M2 activation by IL-4, it was shown in diabetes model that TGFβ1 in combination with high level of glucose resulted in ERK-dependent upregulation of Arg1, suggesting that ERK pathway is involved regulation of Arg1 induced by other M2 stimuli such as TGFβ1 (46). These data support our findings that ERK inhibitor blocked upregulation of Arg1 caused by IL-4. In further support of our data with ERK inhibitors, it was recently shown that anticancer drug puerarin inhibited ERK pathway in TIMs or in IL-4-treated macrophages by skewing balance toward M1 (29). Thus, stimulation of ERK pathway in macrophages appeared to be beneficial for M2-skewing and downmodulation of Th1/17-driven autoimmune inflammation, and *vice versa*, inhibition of ERK pathway is advantageous for M1 skewing in tumor microenvironment and stimulation of anticancer immunity. Taken together, modulation of ERK pathway in macrophages open new possibilities to change M1/M2 balance in various spectrum of disorders ranging from diabetes and cancer to autoimmune disorders and allergy.

ERK pathway is traditionally connected with proliferative capacity of the cells; however, its role in proliferation of macrophages is still not clear (47). It was discovered that tissue-resident M2 macrophages (including CNS-resident microglia during EAE) have high-proliferating capacity *in vivo* when compared with M1 macrophages, but molecular basis for this phenomenon remained not known (14, 34). It was shown that Forskolin-induced proliferation of thioglycollate-elicited macrophages demonstrating important role of CREB in proliferation of macrophages during inflammation (48). Our study linked Forskolin with ERK activation and highlighted importance of ERK pathway in terms of upregulation of expression of Cyclin D1 and enhancement of macrophage proliferation by IL-4. However, we also found that activation of ERK pathway is required but not sufficient to induce and mediate proliferation of M2 macrophages. Further studies will elucidate the role of other pathways besides ERK in this process. One possible candidate is Akt pathway, which is also induced by M-CSF and probably IL-4 (35, 48).

ERK pathway was shown to be important *in vivo* for allergic lung inflammation (33). In EAE model, it was recently shown that inhibition of ERK resulted in downregulation of GM-CSF, a cytokine that promote M1 polarization (49, 50). However, the role of ERK is difficult to estimate *in vivo* during autoimmune neuroinflammation since besides macrophages, ERK inhibitors also affect CD4 T cells and dendritic cells (51, 52). Our study suggests that stimulation of ERK pathway is beneficial during neuroinflammation by changing balance toward M2 in inducing miR-124. We previously demonstrated that miR-124 was very important *in vivo* for EAE and allergic lung inflammation models promoting M2 polarization while not affecting CD4 T cells and dendritic cells (10, 17). Therefore, Forskolin is promising drug that stimulate miR-124 in macrophages and suppress autoimmune neuroinflammation.

The role of cAMP in M2 polarization was recently highlighted in several studies (24, 25, 28). It was also demonstrated that treatment of cultured macrophages with 8-bromo-cAMP together with IL-4 synergistically activated Arg1 promoter (53). We confirmed that cAMP-elevating agent Forskolin also synergized IL-4 to induce 18-fold upregulation of Arg1. Our study also demonstrated for the first time important role of ERK pathway that most likely modulate or serve as a co-factor for other known pathways such as CREB–CEBPβ axis and STAT6 (28, 53). Currently, the role of cAMP in the modulation of ERK activity remains controversial. It was shown that during macrophage development cAMP inhibit M-CSF-induced ERK expression demonstrating negative role in macrophages activation/polarization (54). Our study clearly demonstrate that cAMP activate not only CREB but also ERK, which play an important role in M2 polarization. In addition to CREB, we also found that other members of this family of cAMP-induced transcriptions factors such as ATF3 are upregulated in IL-4-treated M2 macrophages. Quite interesting that ATF3 is downregulated sevenfold by Forskolin as shown for human macrophages (55). Our data also demonstrated that ATF3 was downregulated fivefold by Forskolin in mouse BM-derived macrophages (data not shown). Thus, CREB and ATF3 are regulated by Forskolin in opposite ways. This implies that upregulation of ATF3 in M2 macrophages treated with ERK inhibitors could happen due to compensatory mechanism. In the CNS, ATF3 was upregulated early in EAE during preclinical stage, when few infiltrating leukocytes were migrating into CNS (56), suggesting that ATF3 was most likely induced in microglial cells, which become activated before the onset of EAE (14). Thus, cAMP pathway has multiple actions in M2 skewing of macrophages *in vitro* acting through multiple transcriptional factors, which require further investigation.

The mechanisms how Forskolin skew macrophages toward M2 *in vivo* during EAE is even complex when compared with *in vitro* models since Forskolin affect many different cell types. Macrophages are known to be critical for onset of EAE (57), but this disease is also initiated by autoimmune Th1 and Th17 effector cells (58). Forskolin was shown to inhibit proliferation of T cells *in vitro* during their priming by inhibiting IL-2 signaling (23). In our model, we started administration of Forskolin on the day of disease onset (day 14), when Forskolin affected already primed effector cells. The role of cAMP pathway is more complex in case of effector CD4 T cells, since there are contradictory studies demonstrating either inhibition or stimulation of Th1 and Th17 cells by this pathway (6, 23, 59). Thus, there is a possibility that Forskolin also promote expansion of Th1 and Th17 cells *in vivo* during EAE. If this is the case, we demonstrated that direct action of Forskolin on macrophages could overcome direct effect on T cells. However, what Forskolin was doing with encephalitogenic effector CD4 T cells *in vivo* remained enigmatic, since Forskolin could affect T cells indirectly by modulating function of antigen-presenting cells in the CNS. To address this conundrum, we performed *in vitro* experiment investigating direct effect of Forskolin on the proliferation of primed MOG-specific enriched population of CD4^+^Vβ11^+^ T cells. Our study demonstrated that Forskolin did not have significant effect on proliferation of CD4 T cells that were re-stimulated with MOG peptide. Same results we received when we analyzed MOG-specific enriched population of CD4^+^Vβ11^+^ cells: comparable level of proliferation was observed. On the other hand, we confirmed previously published studies that during polyclonal stimulation of T cells with anti-CD3, Forskolin decreased CD4 T cell proliferation. Our *in vitro* data indicate that MOG-specific autoimmune CD4 T cells were not substantially affected by Forskolin. Thus, we strongly believe that it is unlikely that Forskolin directly inhibits effector autoimmune CD4 T cells during EAE; rather, Forskolin affect macrophages, which result to indirect inhibition of encephalitogenic T cells in the CNS. Since Forskolin did not skew balance toward to M2 in the periphery (spleen) and did not have direct effect of autoimmune CD4 T cells, this likely explains why proliferation and IFNγ production by CD4 T cells in the spleen was not decreased by Forskolin during EAE. We believe that Forskolin affected macrophages in the CNS but not in the spleen is due to the presence of specific CNS microenvironment such as presence of internal source of IL-4 in the CNS (9). Indeed, our data indicate that Forskolin skew macrophages toward M2 in the presence of IL-4 or IL-4 and IFNγ.

In addition to macrophages, Forskolin is known to affect neuronal cells activating BDNF–TrkB–CEBPβ signaling pathway. In neurons, CREB–CEBPβ axis increase survival, repair, and plasticity (59, 60). Finally, Forskolin may affect oligodendrocytes and astrocytes by elevating cAMP and/or ERK1/2 levels and promoting their maturation and differentiation (61–63). We found increased LFB staining in the spinal cords of Forskolin-treated mice in comparison to within unmanipulated (control) mice; this increase could not be attributed to staining artifact. This suggests that Forskolin might improve myelination during recovery from EAE. Further experiments will establish other cell targets for this drug during EAE/MS.

Taken together, our data demonstrated new ERK-dependent mechanism of action of Forskolin in skewing of macrophages toward M2 *in vitro* and *in vivo* during EAE. Our study demonstrate an important role of M1/M2 balance in the CNS for the regulation of autoimmune inflammation and stimulation of pathogenic T cells, which has potential applications for the usage of Forskolin for the therapy of MS and other Th1-mediated autoimmune disorders.

## Ethics Statement

The study was performed in accordance with the recommendations of the ARRIVE guidelines (http://www.nc3rs.org.uk/arrive-guidelines). All animal procedures were conducted under individual licenses from the Department of Health of Hong Kong government and approved by animal ethics committee form the Chinese University of Hong Kong.

## Author Contributions

EP and TV conceived the study. TV and EP designed experiments. TV, AY, MD, IK, and EP conducted experiments. NB and EP performed flow cytometry data analysis. IP, AL, and TS contributed reagents and materials. TV, AY, IP, AL, TS, NB, and EP analyzed the data. TV, IP, AL, TS, NB, and EP prepared the manuscript.

## Conflict of Interest Statement

The authors declare that the research was conducted in the absence of any commercial or financial relationships that could be construed as a potential conflict of interest.

## Supplementary Material

Figure S1 Effect of Forskolin on the activation of downstream signaling pathways CREB, CEBPβ, and ERK1/2 at a baseline level of BM-derived macrophages (Control), or M2 (IL-4) vs. M1 (IFNγ) polarizing conditions. BM-derived macrophages were grown for 5 days in the presence of M-CSF and were treated with Forskolin, or IL4, or IFNγ, or Forskolin/IL4, or Forskolin/IFNγ, or Forskolin/IL4/IFNγ for durations of 10, 20, and 30 min or for 24 h. ERK inhibitor U0126 was used to block ERK1 and ERK2 phosphorylation (see Materials and Methods). Expressions of CREB, CEBPβ, and ERK1/2 and their phosphorylated forms (p-CREB, p-CEBPβ, and p-ERK1/2) were analyzed by Western blot as described in Section “Materials and Methods.” β-Actin was used as a loading control. The same stripped membrane was used to evaluate the expression of all proteins including β-actin. These data are representative of three separate experiments (abbreviations: I, IFNγ; 4, IL-4; F, Forskolin; U, U0126).

Figure S2 *In silico* analysis of the presence of cAMP-response elements (CRE) in promoter areas of microRNA (miR)-124 precursor molecule pre-miR-124-3 and potential transcription factors, which upregulate miR-124 expression in macrophages. **(A)** Mapping of cAMP-response elements within 2,000 bp promoter region upstream of miR-124 precursor molecule pre-miR-124-3. A list of transcription factors that could potentially bind CRE sites are shown on the right. Two selected transcription factors CREB and activating transcription factor (ATF)3 are marked in red. Similar CRE sites for binding of CREB/ATF family transcriptions factors were also found for pre-miR-124-2 but not pre-miR-124-1 precursor molecule (data not shown). **(B)** Promoter area for pre-miR-124-3 with CRE sites are shown for mouse chromosome 2 and human chromosome 20.

Figure S3 Influence of activating transcription factor (ATF)3 activator taurolidin on microRNA (miR)-124 expression in bone marrow (BM)-derived macrophages. BM-macrophages were treated with taurolidin for 24 h and miR-124 expression was analyzed as described in Section “Materials and Methods.” Mean ± SE of triplicate is shown (***p* < 0.01).

Figure S4 The role of RE1-silencing transcription factor (REST) in the expression of microRNA (miR)-124 in macrophages. Bone marrow (BM)-derived macrophages were cultured for 5 days as described in Section “Materials and Methods.” Then, samples that were untreated (Control), treated with Forskolin **(A,B)**, or treated with REST inhibitors **(C,D)** for the indicated periods were analyzed for the expression of REST **(A,B)** or miR-124 **(C,D)**. **(A)** Analysis of the expression of REST and REST4 in BM-derived macrophages on the mRNA level by RT-PCR. BM-derived macrophages were analyzed as untreated (Control) or treated with Forskolin (FSK) for 2–4 h. Mouse primary cortical neurons (Neu) were used as a control with a low level of REST and REST4 expressions. Negative control (Neg) was a medium. MW indicate molecular weight marker (in bp). GADPH loading control is shown below. **(B)** Analysis of the modulation of the expression of REST in BM-derived macrophages that were untreated for 4 or 24 h or treated with Forskolin for 4 or 24 h. Expressions of REST were analyzed with Western blot as described in Section “Materials and Methods.” Actin was used as a loading control. Representative image is shown on the left, quantitative analysis of expression of REST normalized do β-actin is shown on the right. Mean ± SE of three separate experiments is shown. **(C)** BM-derived macrophages were incubated with REST inhibitor X5050 for 6 or 16 h (overnight), after which the expression of miR-124 was assessed by real-time RT-PCR as described in Section “Materials and Methods.” **(D)** BM-derived macrophages were incubated with REST inhibitory dsRNA NRSE for 16 h, after which expression of miR-124 was assessed by real-time RT-PCR as described in Section “Materials and Methods.” REST inhibitory dsRNA NRSE resulted in 1.5-fold upregulation of miR-124. Mean ± SE of triplicate is shown (**p* < 0.05).

## Abbreviations

Arg, arginase; ATF, activating transcription factor; BM, bone marrow; CNS, central nervous system; CRE, cAMP response elements; EAE, experimental autoimmune encephalomyelitis; LFB, luxol fast blue; MOG, myelin oligodendrocyte glycoprotein; Mrc, mannose receptor; miR, microRNA; MS, multiple sclerosis; NOS, nitric oxide synthetase; REST, RE1-silencing transcription factor.
