# Supplementary figures and images for: Cyclic AMP Pathway Suppress Autoimmune Neuroinflammation by Inhibiting Functions of Encephalitogenic CD4 T Cells and Enhancing M2 Macrophage Polarization at the Site of Inflammation
Source: Front Immunol. 2018 Jan 25;9:50. doi: 10.3389/fimmu.2018.00050 (PMC5788911; doi:10.3389/fimmu.2018.00050)

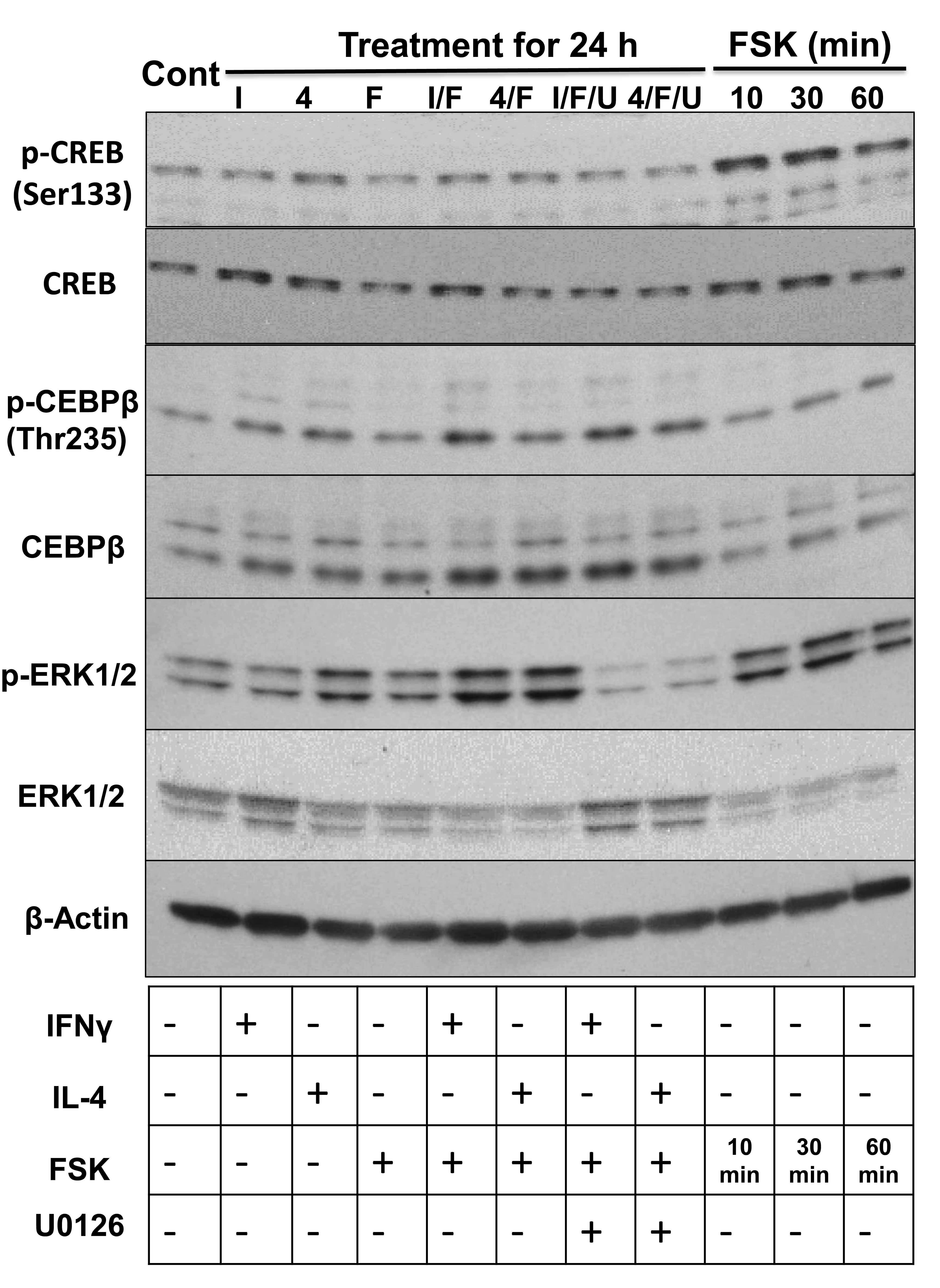

Supplement: Figure S1 — Effect of Forskolin on the activation of downstream signaling pathways CREB, CEBPβ, and ERK1/2 at a baseline level of BM-derived macrophages (Control), or M2 (IL-4) vs. M1 (IFNγ) polarizing conditions. BM-derived macrophages were grown for 5 days in the presence of M-CSF and were treated with Forskolin, or IL4, or IFNγ, or Forskolin/IL4, or Forskolin/IFNγ, or Forskolin/IL4/IFNγ for durations of 10, 20, and 30 min or for 24 h. ERK inhibitor U0126 was used to block ERK1 and ERK2 phosphorylation (see Materials and Methods). Expressions of CREB, CEBPβ, and ERK1/2 and their phosphorylated forms (p-CREB, p-CEBPβ, and p-ERK1/2) were analyzed by Western blot as described in Section “Materials and Methods.” β-Actin was used as a loading control. The same stripped membrane was used to evaluate the expression of all proteins including β-actin. These data are representative of three separate experiments (abbreviations: I, IFNγ; 4, IL-4; F, Forskolin; U, U0126). [file Image_1.jpg]

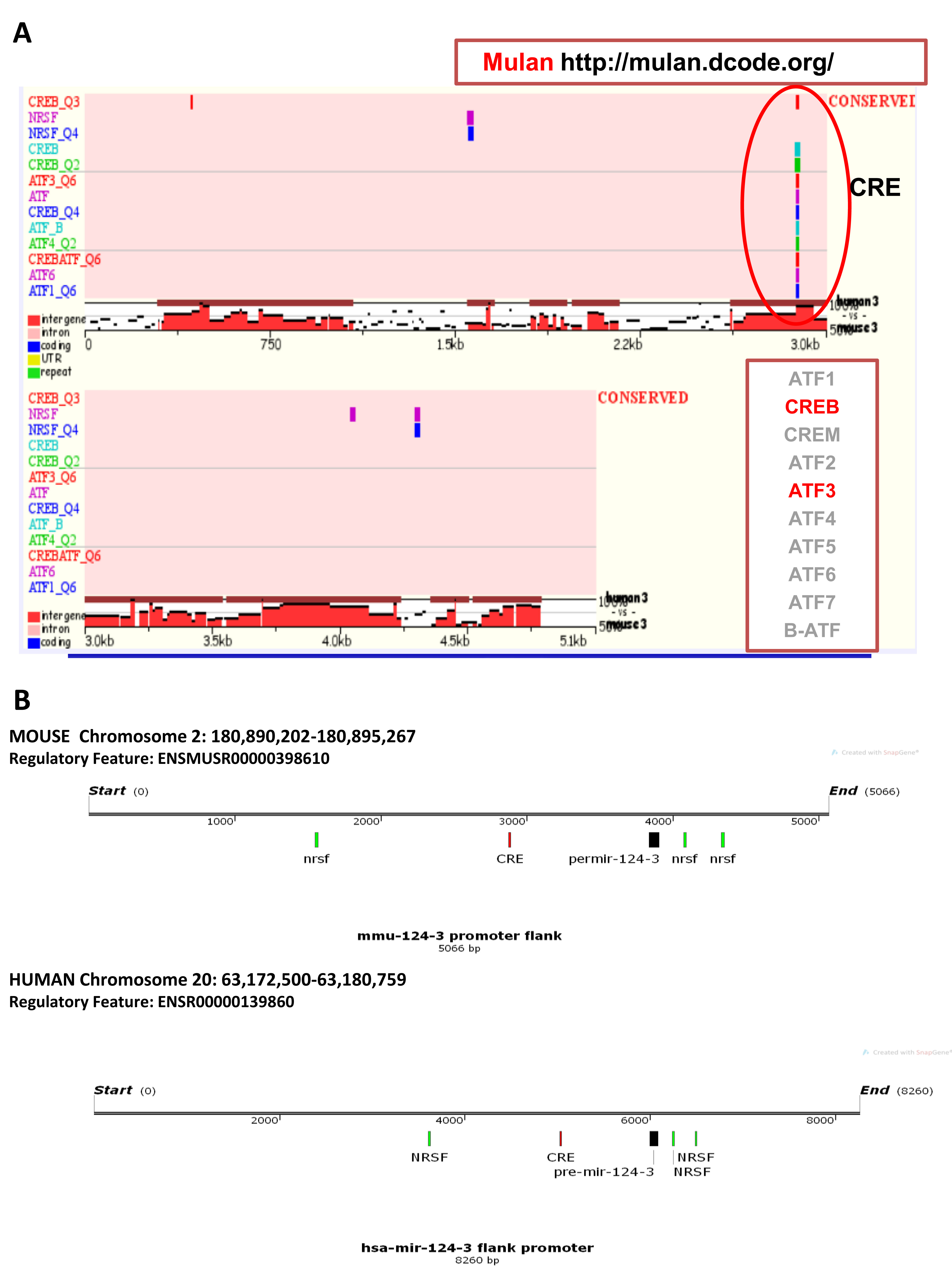

Supplement: Figure S2 — In silico analysis of the presence of cAMP-response elements (CRE) in promoter areas of microRNA (miR)-124 precursor molecule pre-miR-124-3 and potential transcription factors, which upregulate miR-124 expression in macrophages. (A) Mapping of cAMP-response elements within 2,000 bp promoter region upstream of miR-124 precursor molecule pre-miR-124-3. A list of transcription factors that could potentially bind CRE sites are shown on the right. Two selected transcription factors CREB and activating transcription factor (ATF)3 are marked in red. Similar CRE sites for binding of CREB/ATF family transcriptions factors were also found for pre-miR-124-2 but not pre-miR-124-1 precursor molecule (data not shown). (B) Promoter area for pre-miR-124-3 with CRE sites are shown for mouse chromosome 2 and human chromosome 20. [file Image_2.jpg]

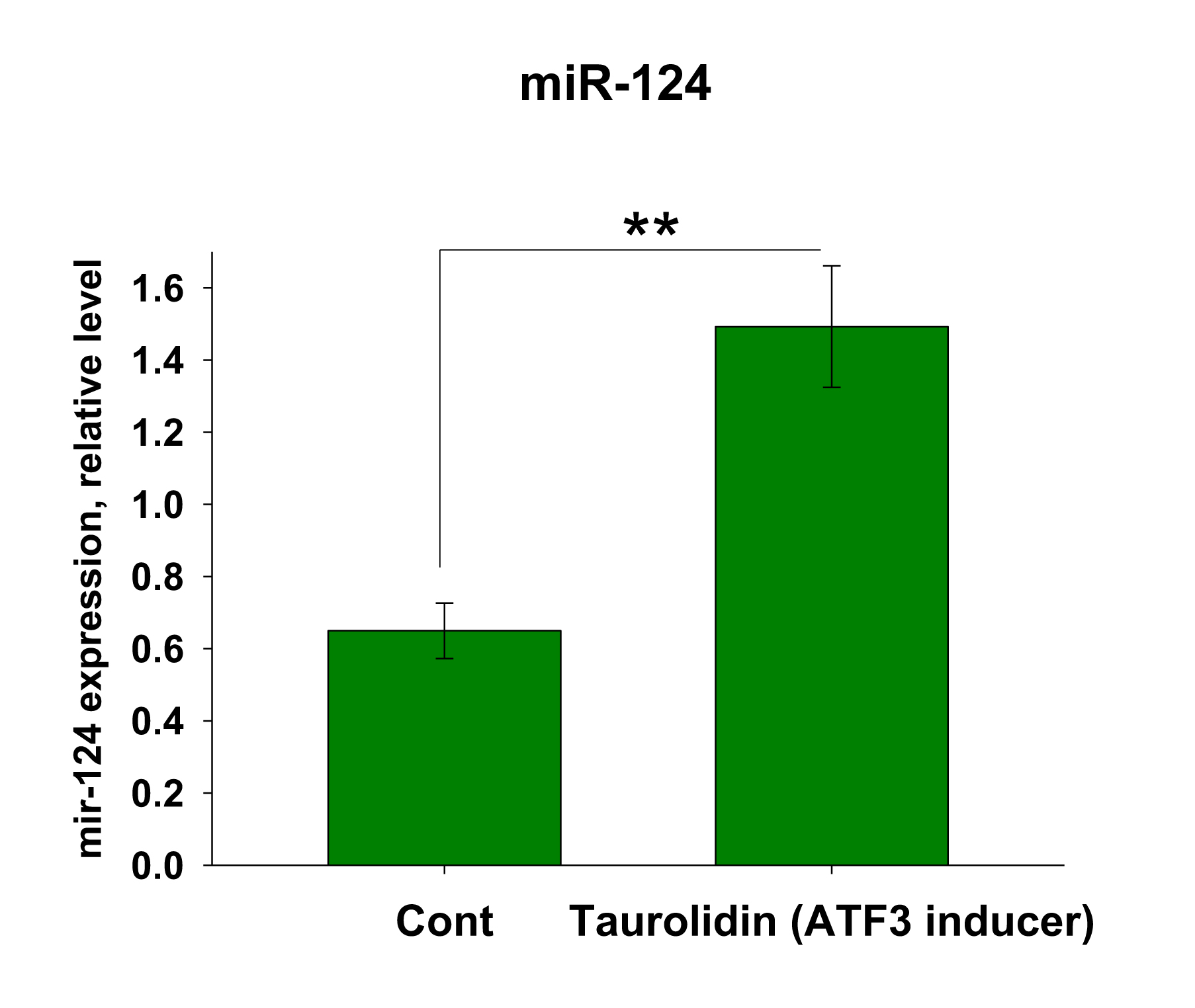

Supplement: Figure S3 — Influence of activating transcription factor (ATF)3 activator taurolidin on microRNA (miR)-124 expression in bone marrow (BM)-derived macrophages. BM-macrophages were treated with taurolidin for 24 h and miR-124 expression was analyzed as described in Section “Materials and Methods.” Mean ± SE of triplicate is shown (**p < 0.01). [file Image_3.jpg]

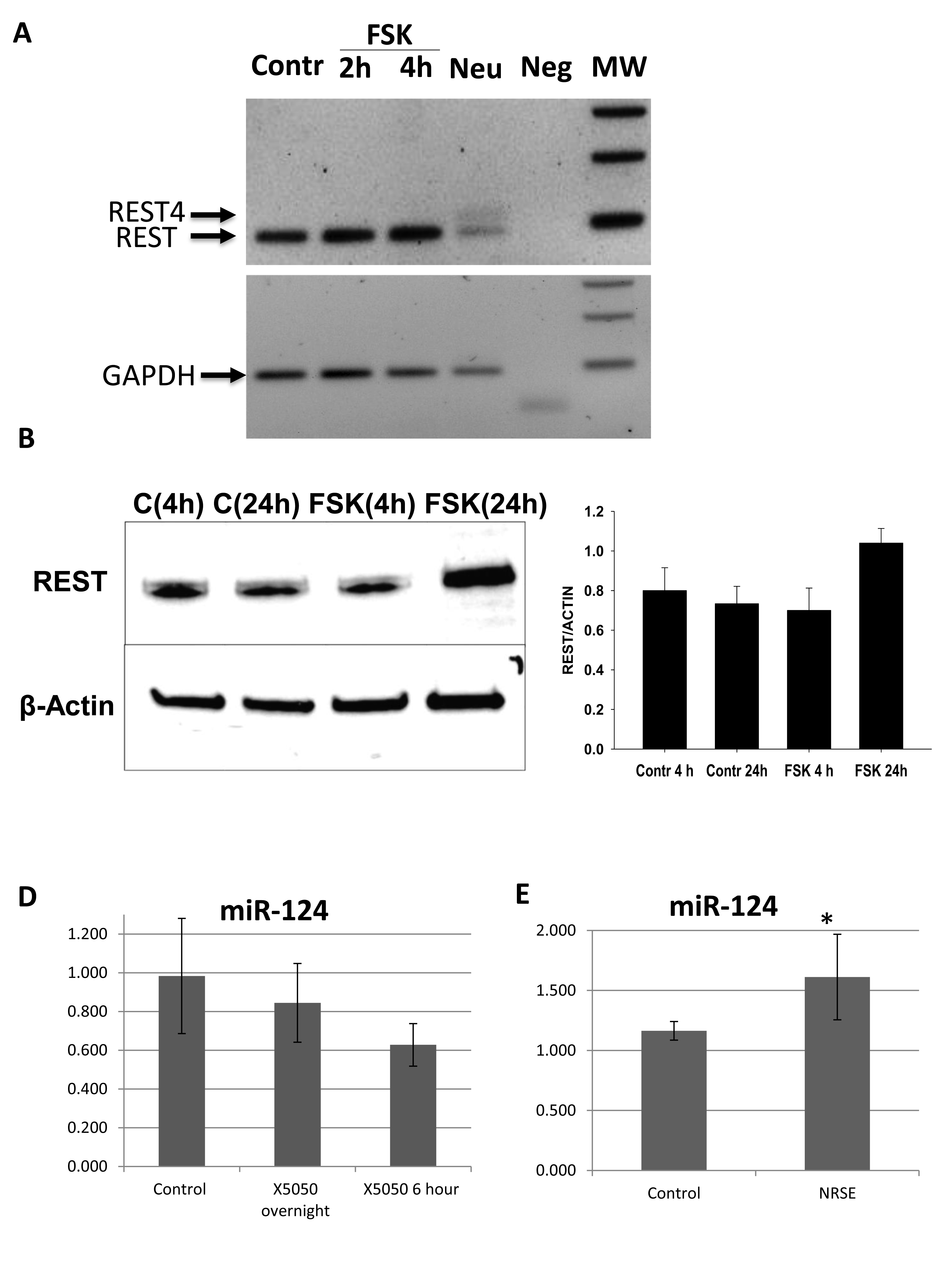

Supplement: Figure S4 — The role of RE1-silencing transcription factor (REST) in the expression of microRNA (miR)-124 in macrophages. Bone marrow (BM)-derived macrophages were cultured for 5 days as described in Section “Materials and Methods.” Then, samples that were untreated (Control), treated with Forskolin (A,B), or treated with REST inhibitors (C,D) for the indicated periods were analyzed for the expression of REST (A,B) or miR-124 (C,D). (A) Analysis of the expression of REST and REST4 in BM-derived macrophages on the mRNA level by RT-PCR. BM-derived macrophages were analyzed as untreated (Control) or treated with Forskolin (FSK) for 2–4 h. Mouse primary cortical neurons (Neu) were used as a control with a low level of REST and REST4 expressions. Negative control (Neg) was a medium. MW indicate molecular weight marker (in bp). GADPH loading control is shown below. (B) Analysis of the modulation of the expression of REST in BM-derived macrophages that were untreated for 4 or 24 h or treated with Forskolin for 4 or 24 h. Expressions of REST were analyzed with Western blot as described in Section “Materials and Methods.” Actin was used as a loading control. Representative image is shown on the left, quantitative analysis of expression of REST normalized do β-actin is shown on the right. Mean ± SE of three separate experiments is shown. (C) BM-derived macrophages were incubated with REST inhibitor X5050 for 6 or 16 h (overnight), after which the expression of miR-124 was assessed by real-time RT-PCR as described in Section “Materials and Methods.” (D) BM-derived macrophages were incubated with REST inhibitory dsRNA NRSE for 16 h, after which expression of miR-124 was assessed by real-time RT-PCR as described in Section “Materials and Methods.” REST inhibitory dsRNA NRSE resulted in 1.5-fold upregulation of miR-124. Mean ± SE of triplicate is shown (*p < 0.05). [file Image_4.jpg]
